# Antiviral, anti-inflammatory and antioxidant effects of curcumin and curcuminoids in SH-SY5Y cells infected by SARS-CoV-2

**DOI:** 10.1038/s41598-024-61662-7

**Published:** 2024-05-10

**Authors:** Tiago Nicoliche, Cynthia Silva Bartolomeo, Robertha Mariana Rodrigues Lemes, Gabriela Cruz Pereira, Tamires Alves Nunes, Rafaela Brito Oliveira, Arthur Luiz Miranda Nicastro, Érica Novaes Soares, Brenno Fernandes da Cunha Lima, Beatriz Moreira Rodrigues, Juliana Terzi Maricato, Liria Hiromi Okuda, Mirela Inês de Sairre, Carla Máximo Prado, Rodrigo Portes Ureshino, Roberta Sessa Stilhano

**Affiliations:** 1 Department of Physiological Sciences, Santa Casa de São Paulo School of Medical Sciences (FCMSCSP), 61 Dr. Cesário Mota Junior Street, São Paulo, SP 01221-020 Brazil; 2 Department of Bioscience, Federal University of São Paulo (UNIFESP), São Paulo, Brazil; 3 Department of Biological Sciences, Federal University of São Paulo (UNIFESP), São Paulo, Brazil; 4 Post-Graduation Program in Chemistry-Biology, Federal University of São Paulo (UNIFESP), Diadema, Brazil; 5 Department of Biochemistry, Federal University of São Paulo (UNIFESP), São Paulo, Brazil; 6 Federal University of Bahia (UFBA), Bahia, Brazil; 7 Department of Microbiology, Immunology and Parasitology, Federal University of São Paulo (UNIFESP), São Paulo, Brazil; 8 Biological Institute, Agriculture and Supply Department, São Paulo, SP Brazil; 9 Human and Natural Sciences Center, Federal University of ABC (UFABC), São Paulo, Brazil

**Keywords:** COVID19, Curcumin, Oxidative stress, Neuroinflammation, Neuronal cell, Cell death, Cell death in the nervous system, Natural products, Cell biology, Plant sciences

## Abstract

COVID-19, caused by SARS-CoV-2, affects neuronal cells, causing several symptoms such as memory loss, anosmia and brain inflammation. Curcuminoids (Me08 e Me23) and curcumin (CUR) are derived from *Curcuma Longa extract* (EXT). Many therapeutic actions have been linked to these compounds, including antiviral action. Given the severe implications of COVID-19, especially within the central nervous system, our study aims to shed light on the therapeutic potential of curcuminoids against SARS-CoV-2 infection, particularly in neuronal cells. Here, we investigated the effects of CUR, EXT, Me08 and Me23 in human neuroblastoma SH-SY5Y. We observed that Me23 significantly decreased the expression of plasma membrane-associated transmembrane protease serine 2 (TMPRSS2) and TMPRSS11D, consequently mitigating the elevated ROS levels induced by SARS-CoV-2. Furthermore, Me23 exhibited antioxidative properties by increasing *NRF2* gene expression and restoring NQO1 activity following SARS-CoV-2 infection. Both Me08 and Me23 effectively reduced SARS-CoV-2 replication in SH-SY5Y cells overexpressing ACE2 (SH-ACE2). Additionally, all of these compounds demonstrated the ability to decrease proinflammatory cytokines such as IL-6, TNF-α, and IL-17, while Me08 specifically reduced INF-γ levels. Our findings suggest that curcuminoid Me23 could serve as a potential agent for mitigating the impact of COVID-19, particularly within the context of central nervous system involvement.

## Introduction

The emergence of the novel coronavirus, SARS-CoV-2, has had a profound and lasting impact, resulting in a significant global burden of morbidity and mortality. To date, > 670,000,000 people have been infected, resulting in > 6,800,000 deaths globally (https://coronavirus.jhu.edu/map.html, accessed on 03 October 2023). Beyond the acute phase of the disease, the pandemic has also raised concerns regarding its lingering effects, particularly neurological dysfunction. Over the past three years, it has become increasingly evident that a substantial proportion of individuals afflicted by SARS-CoV-2, estimated at 10–15%, continue to experience a range of post-COVID-19 symptoms^1^. This condition, often referred to as Long-COVID syndrome, manifests with a diverse array of symptoms, including fatigue, hyposmia, shortness of breath, headache, disturbances in the sense of smell or taste, myalgia, anxiety, and cognitive deficits, commonly described as "brain fog," encompassing memory impairment and other cognitive symptoms^2–7^. Notably, individuals who suffered severe manifestations of SARS-CoV-2 infection are at greater risk of developing neurological symptoms, with some even experiencing conditions like viral encephalitis, necrotizing encephalopathy, acute myelitis, or Guillain-Barré syndrome^8–12^. However, the exact neuropathogenesis underlying SARS-CoV-2-related neurological conditions remains unclear, particularly in distinguishing direct and indirect effects of the virus itself.

These post-infection neurological symptoms are closely tied to host cell factors that play pivotal roles in the early stages of viral entry. These factors are crucial determinants of coronavirus tropism and the efficiency of cellular entry^13–15^. The initial attachment of the virus to the target cell hinges on the interaction between the viral spike (S) protein and the host receptor, angiotensin-converting enzyme 2 (ACE2)^14,16^. Additionally, various proteases, including furin, cathepsin B/L, the plasma membrane-associated transmembrane proteases serine 2 (TMPRSS2), TMPRSS11D, and TMPRSS13, actively participate in these processes^14–17^. Consequently, the expression levels of these key proteins become essential factors in the SARS-CoV-2 tropism.

Moreover, oxidative stress and neuroinflammation have emerged as potential contributors to the neurological symptoms observed in COVID-19. High levels of reactive oxygen species (ROS) are a common feature of chronic conditions and viral infections, including COVID-19^18^. During the infection of host cells, coronaviruses trigger an imbalance characterized by heightened ROS production and weakened antioxidant host responses, culminating in increased redox stress^19^. ROS may initiate and exacerbate inflammatory responses by engaging specific signaling pathways, such as the NF-κB pathway^20,21^. NF-κB regulates numerous genes involved in inflammatory responses, including proinflammatory cytokines (e.g., IL-1β, IL-6, and TNF-α), resulting in their increased expression in tissues and consequent direct cytotoxic effects^22^. Reports have demonstrated the upregulation of proinflammatory cytokines, such as IL-1β, IL-6, and TNF-α, following ischemic events and intracerebral hemorrhages, leading to neuroinflammation^23,24^. These cytokines serve as pivotal mediators of inflammatory responses, and their overexpression can further activate downstream apoptotic signaling pathways in neurons, ultimately culminating in neuronal cell death^25^.

Curcumin (CUR), a natural spice, has garnered significant attention for its potential in treating conditions characterized by immune system perturbations and inflammatory responses, including COVID-19^26–29^. CUR and other curcuminoids are the primary bioactive components of turmeric (*Curcuma longa*), a substance that has been employed in the traditional medicine practices of diverse cultures for centuries ^30,31^. This enduring use is attributed to the anti-inflammatory, antioxidant, antibacterial, antiviral and neuroprotective properties exhibited by curcuminoids^32–34^. Furthermore, curcuminoids can play a role in inhibiting certain enzymes associated with the inflammatory process, such as mitogen-activated protein kinases (MAPKs), c-Jun N-terminal kinases (JNK), and nuclear factor kappa B (NF-kB)^35^. The antioxidant properties of curcuminoids have been shown to inhibit carcinogenic reactive oxygen species (ROS), including superoxide anions, hydroxyl radicals, nitrites, and peroxides^36^.One of the most significant enzymes involved in the antioxidative pathway is NAD(P)H:quinone oxidoreductase 1 (NQO1), which catalyzes the two-electron reduction of various endogenous and exogenous quinones using flavin adenine nucleotides (FAD) as a cofactor. The NQO1 gene contains antioxidant response element (ARE) sequences in its promoter region and has been demonstrated to be regulated by nuclear factor (erythroid-derived 2)-like 2 (NRF2)^37^. Curcumin can stimulate both the activation of antioxidant enzymes and the decrease in expression of pro-inflammatory cytokines such as MCP-1, TNF-a, IL-6 e IL-1b through the activation of the NRF2 pathway^38,39^.

A recent systematic review identified six studies demonstrating that curcumin supplementation led to a significant decrease in common COVID-19 symptoms, reduced hospitalization duration, and decreased mortality rates^40^. The authors concluded that curcumin treatment mitigates the manifestation of cytokine storms by reducing pro-inflammatory factors and activating anti-inflammatory pathways^40^. They further suggested that curcumin treatment may alleviate COVID-19 symptoms by restoring the balance between pro-inflammatory and anti-inflammatory responses.

Despite existing studies highlighting the antiviral effects of curcumin against SARS-CoV-2, there remains a conspicuous gap in the literature regarding its antiviral effects in both in vitro and in vivo neuronal models infected with SARS-CoV-2. Furthermore, the emergence of novel curcuminoids, exemplified by Me08 and Me23, characterized by their simple chemical structures and remarkable stability, with promising antiproliferative properties^41–43^, presents an uncharted avenue in the context of COVID-19 research.

In light of these considerations, our current study explores the potential antiviral, antioxidant, and anti-inflammatory effects of CUR, EXT, and curcuminoids Me08 and Me23 in the SH-SY5Y neuronal cell line. Through this research, we aim to shed light on the potential therapeutic value of curcuminoids in the context of SARS-CoV-2 infection, particularly within neuronal cell models.

## Results

### Cytotoxicity of CUR, EXT and curcuminoids on SH-SY5Y cells

**Figure 1:**
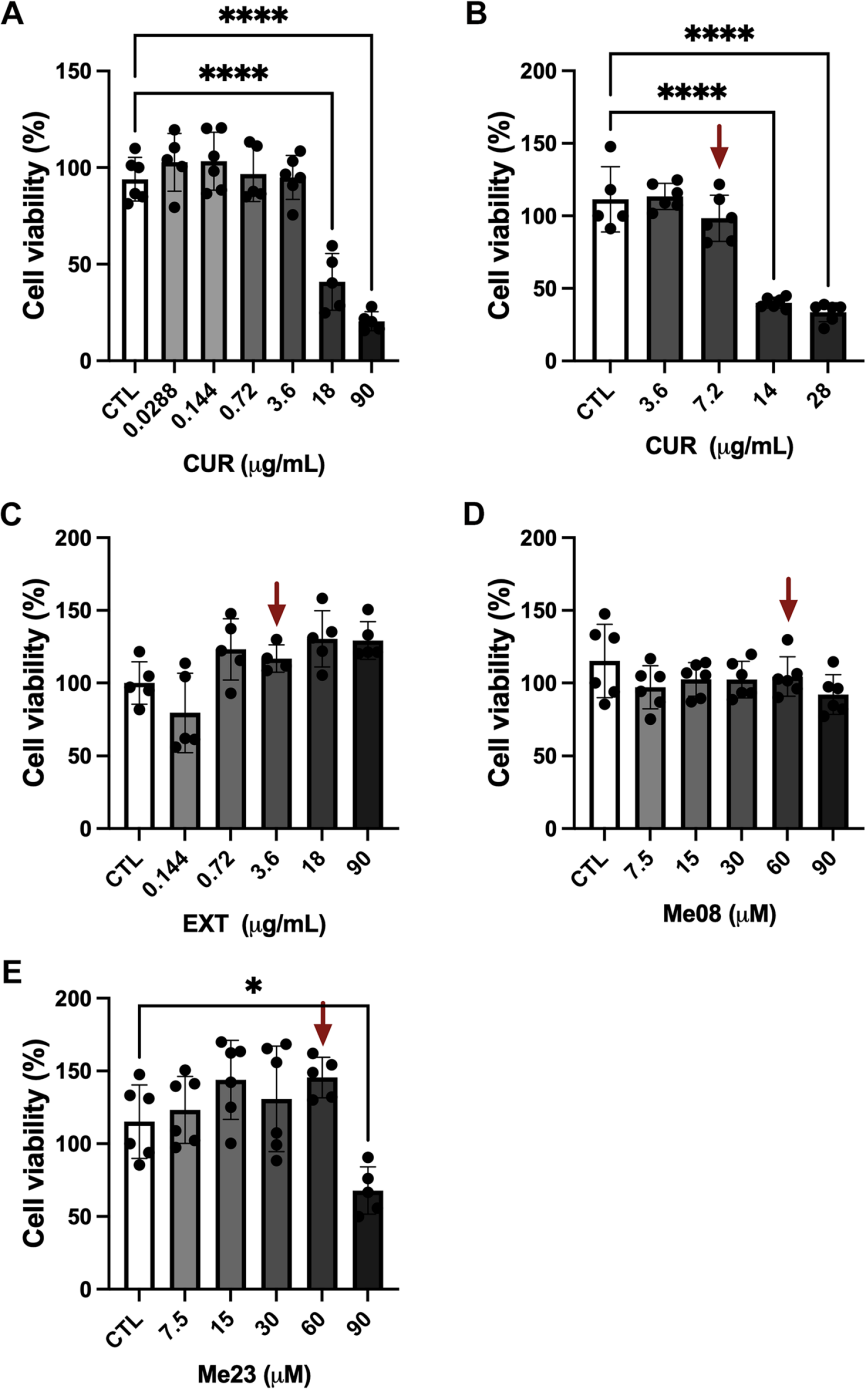
Cytotoxic of CUR, EXT, Me08 and Me23 on SH-SY5Y. Cells were seeded on day 0 and treated after 24 h (Day 1), the MTT was performed 24 h after treatment (Day 2). (**A**) CUR (90–0.0288 μg/mL), (**B**) CUR (28–3.6 μg/mL), (**C**) EXT (90–0.144 μg/mL), (**D**) Me08 (90–7.5 μM), (**E**) Me23 (90–7.5 μM). *p < 0.05, ****p < 0.0001. (n = 5–6). Red arrow represents the concentration used in the experiments.

To investigate the cytotoxic effects of CUR, EXT (Extract) and curcuminoids on SH-SY5Y cells, we treated the cells with different concentrations of the compounds and performed a MTT assay after 24 h. We observed that CUR exhibited cytotoxicity at concentrations of 14, 18, 28, and 90 µg/mL, resulting in cell viabilities of 40.1%, 40.88%, 33.4%, and 20.46%, respectively (Fig. 1A,B, One-way ANOVA, F_6,31_ = 35.72, p < 0.0001). Conversely, the viability of SH-SY5Y cells remained at or above 95% when exposed to CUR concentrations of 7.2 µg/mL or lower (Fig. 1A,B). Consequently, we decided to use the non-toxic higher concentration of CUR, which was 7.2 µg/mL, in our future experiments. In contrast, EXT did not exhibit cytotoxicity at any of the concentrations examined. We chose the concentration of 3.6 µg/mL, the highest concentration that maintained viability similar to the control (CTL) (Fig. 1C), for our subsequent experiments. Regarding the curcuminoids Me08 and Me23, no toxicity was observed at concentrations below 60 µM (Fig. 1D,E). However, Me23 exhibited cytotoxicity at 90 µM (cell viability of 67.78%, One-way ANOVA, F_5,28_ = 6.588, p = 0.0004), while the same concentration was non-toxic for Me08. Consequently, we chose the concentration of 60 µM for our future experiments with the curcuminoids.

### Me23 reduces proteases expression in SH-SY5Y cells

**Figure 2:**
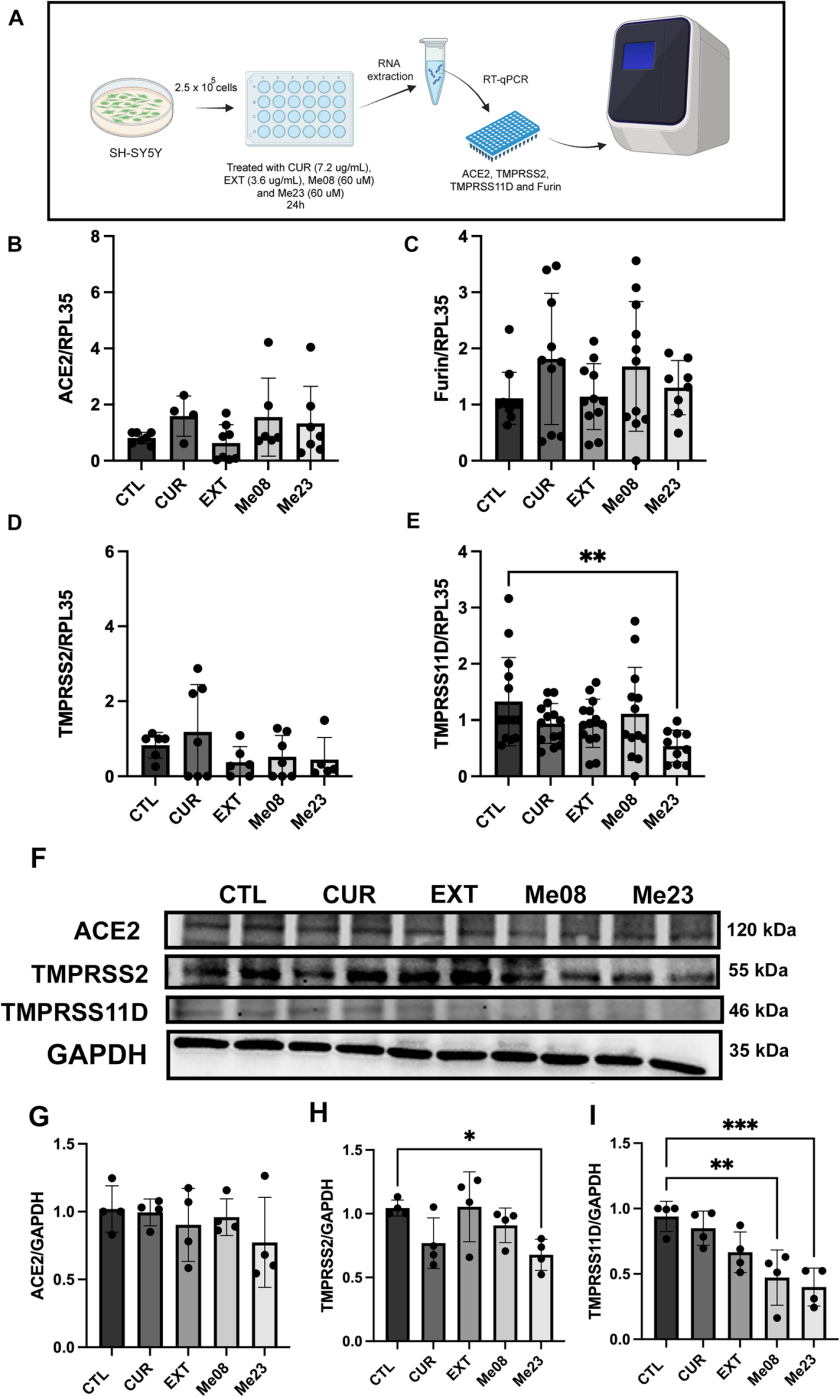
SARS-CoV-2 receptors expression in SH-SY5Y. Cells were treated with CUR, EXT, Me08 or Me23 for 24 h and the gene expression was analyzed by RT-qPCR and protein expression was analyzed by Western-blotting. Gene expression of (**A**) ACE2 (**B**) Furin, (**C**) TMPRSS2, (**D**) TMPRSS11D. Gene expression was normalized by the endogenous expression of RPL35. (**E**) Protein expression according to western blot analyses. (**F**) Histograms reporting the mean ± SD of (**G**) ACE2, (**H**) TMPRSS2, (**I**) TMPRSS11D levels after normalization with the average intensity of the bands from four independent experiments. Protein expression was normalized by the endogenous expression of GAPDH. CTL: cells without treatment, CUR: curcumin, EXT: extract. *p < 0.05. (n = 4–13). The entire blots are presented in Supplementary Figure S1.

To evaluate the effect of compounds CUR (7.2 µg/mL), EXT (3.6 µg/mL), Me08 (60 µM), and Me23 (60 µM) on the gene expression levels of *ACE2*, *Furin*, *TMPRSS2*, and *TMPRSS11D*, which play crucial roles in the entry of SARS-CoV-2 into host cells, we treated SH-SY5Y cells with these compounds for 24 h. Subsequently, we conducted RT-qPCR analysis (Fig. 2A). We did not observe statistically significant differences in the expression of ACE2 (Fig. 2B, One-way ANOVA, F_4,27_ = 1.331, p = 0.2840), Furin (Fig. 2C, One-way ANOVA, F_4,46_ = 1.502, p = 0.2173), or TMPRSS2 (Fig. 2D, One-way ANOVA, F_4,26_ = 1.334, p = 0.2838) following treatment with the compounds. However, it is important to highlight that TMPRSS11D expression exhibited a twofold downregulation when compared to the CTL without treatment (Fig. 2E, One-way ANOVA, F_4,60_ = 2.812, p = 0.0331). We also investigated the compounds’ effect on the protein expression levels of ACE2, TMPRSS2 and TMPRSS11D (Fig. 2F). No changes in ACE2 levels were observed (Fig. 2G, One-way ANOVA, F_4,15_ = 0.7942, p = 0.7942). However, Me23 reduced TMPRSS2 levels by 30% (Fig. 2H, One-way ANOVA, F_4,15_ = 3.662, p = 0.0284). TMPRSS11D protein levels were also reduced by 50% and 60%, respectively after treatment with Me08 and Me23 (Fig. 2I, One-way ANOVA, F_4,15_ = 9.030, p = 0.0006).

### ME23 reduces ROS induced by SARS-CoV-2

**Figure 3:**
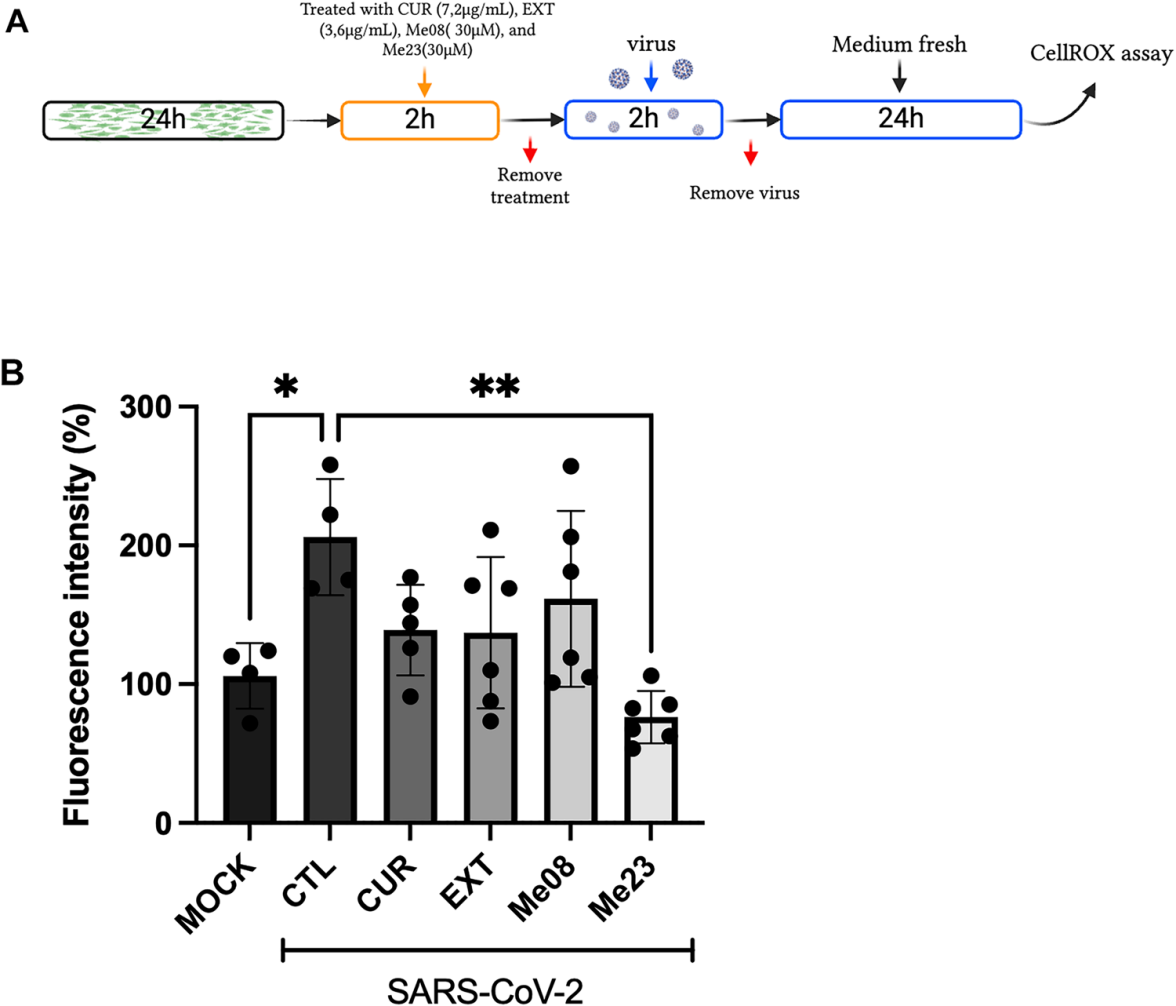
ROS quantification after treatment with CUR, EXT, Me08, Me23. Cells were treated with CUR, EXT, Me08 or Me23 for 2 h, after, they were infected with SARS-CoV-2 (MOI 0.2) for 24 h and the ROS was measured by flow cytometry. The percentage of fluorescence was compared to the MOCK. *p < 0.05. (n = 4–6). The schematic diagram illustrating the infection process was based on a previous publication^67^.

To assess the potential of the compounds to mitigate ROS production following SARS-CoV-2 infection in SH-SY5Y cells, we infected the cells with the virus. After a 2-h incubation with SARS-CoV-2, we removed the virus and allowed the cells to incubate for an additional 24 h only with media (Fig. 3A). Basal ROS levels were quantified in MOCK cells, which served as the reference set at 100%. Cells infected with SARS-CoV-2 but left untreated were designated as the CTL.

The ROS levels in the CTL group were significantly elevated compared to the MOCK cells, with an increase of 206 ± 42% (Fig. 3B, One-way ANOVA, F_5,25_ = 5.053, p < 0.05). However, when cells were treated with Me23, these elevated ROS levels were notably reduced to 76 ± 21% (Fig. 3B, One-way ANOVA, F_5,25_ = 5.053, p = 0.0025). This finding shows the ability of Me23 to mitigate oxidative stress in neuronal cells.

### Me23 demonstrates antioxidant effects following SARS-CoV-2 infection

**Figure 4:**
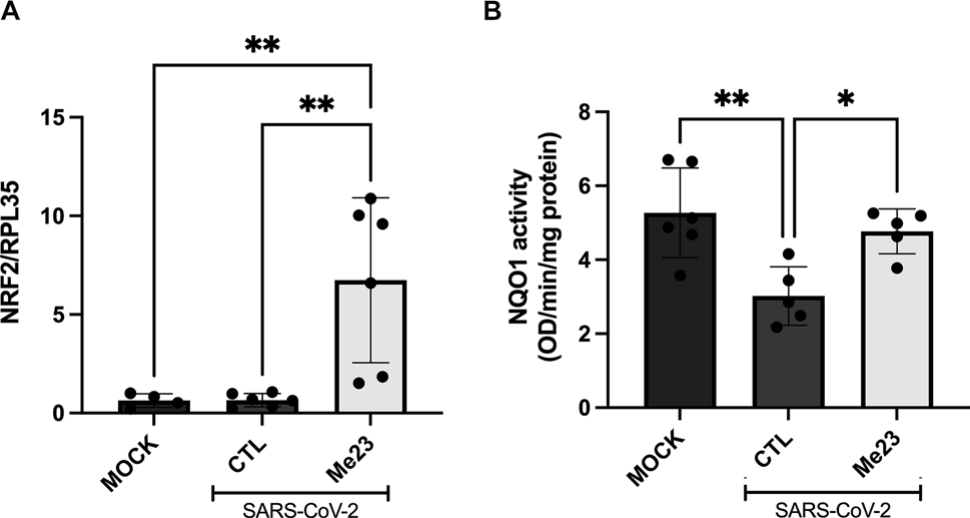
NRF2 expression and NQO1 activity after treatment with Me23. Cells were treated with Me23 for 2 h, after, they were infected with SARS-CoV-2 (MOI 0.2) for 24 h and the *NRF2* gene expression and NQO1 activity was evaluated. (**A**) *NRF2* gene expression was evaluated by qRT-PCR. (**B**) NQO1 activity. **p < 0.005. (n = 4–6). MOCK: SH-SY5Y cells not infected with SARS-CoV-2.

Based on previous findings indicating the antioxidant effects of CUR or curcuminoids via the NRF2 pathway, we decided to investigate the expression of this gene following SARS-CoV-2 infection and treatment with Me23, the only compound capable of reducing ROS levels (Fig. 3B). Notably, SARS-CoV-2 infection did not change the gene expression of *NRF2*. However, treatment with Me23 resulted in an approximately tenfold increase in *NRF*2 expression compared to the CTL and MOCK groups (Fig. 4A, One-way ANOVA, F_2,13_ = 10.23, p = 0.0021). Since NRF2 upregulates NQO1, we also assessed NQO1 activity. SARS-CoV-2 infection reduced NQO1 activity by 1.7-fold (Fig. 4B, One-way ANOVA, F_5,25_ = 8.440, p = 0.0045, however, in the presence of Me23, the activity was restored to levels similar to those observed in the MOCK.

### ACE2 Overexpression in SH-SY5Y Cells reveal the antiviral effects of Curcuminoids Me08 and Me23

**Figure 5:**
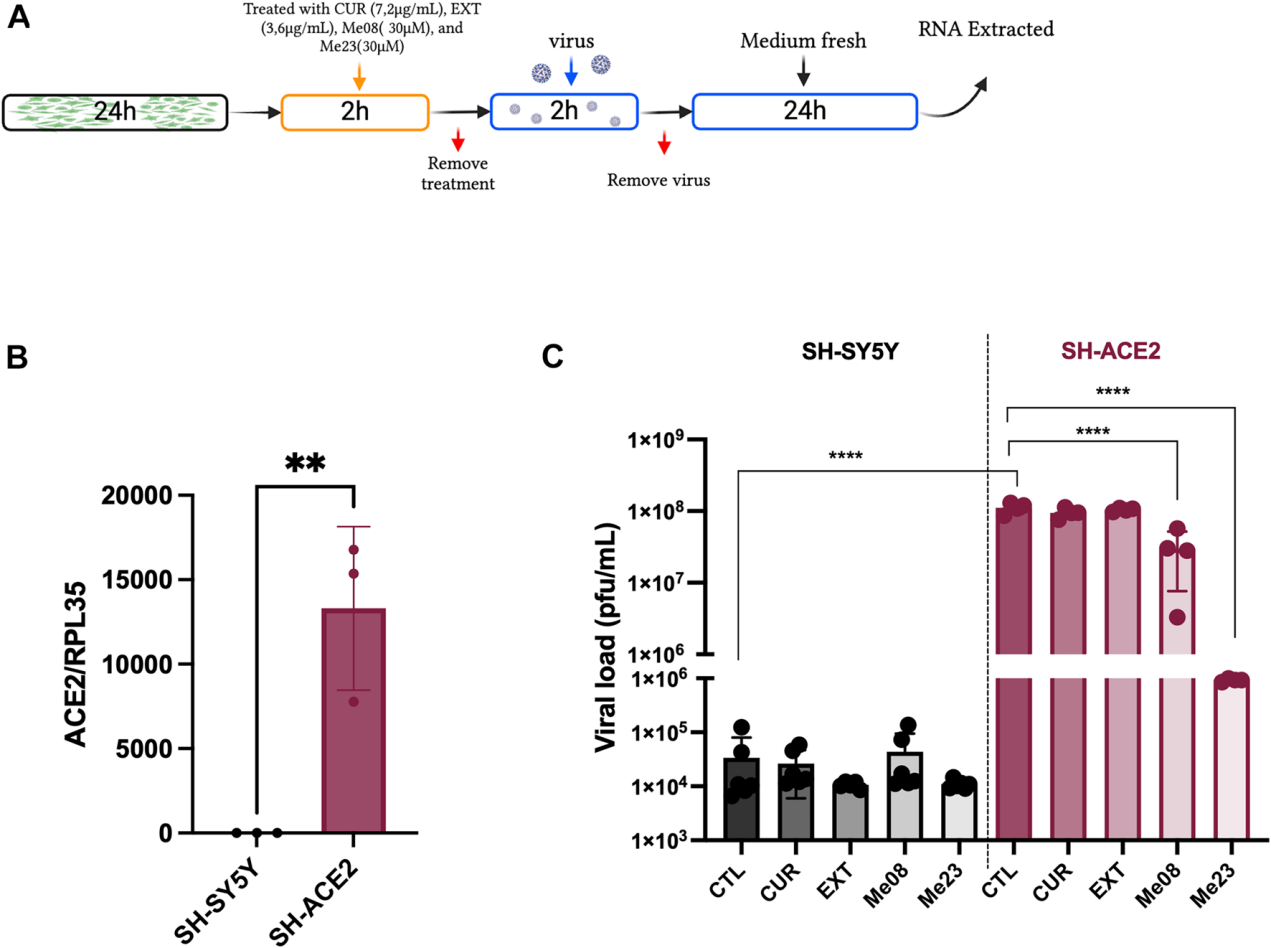
ACE2 overexpression increases viral load in SH-SY5Y and potentialize the curcuminoids effect. (**A**) SH-SY5Y were transduced with a lentivector carrying the ACE2 gene and the cell line SH-ACE2 was created. The overexpression of ACE2 was evaluated by RT-qPCR. (**B**) SH-SY5Y and SH-ACE2 were treated with CUR, EXT, Me08 or Me23 for 2 h and after they were infected with SARS-CoV-2 (MOI 0.2) for 24 h. (**C**) The viral load was quantified by RTqPCR. CTL: Cells infected with SARS-CoV-2 and without treatment, CUR: curcumin, EXT: extract. **p < 0.01, ****p < 0.0001. (n = 3–6). The schematic diagram illustrating the infection process was based on a previous publication^67^.

Considering previous publications indicating that SH-SY5Y cells exhibit low levels of SARS-CoV-2 replication^14^, we have decided to overexpress ACE2 to bolster viral replication within these cells. To achieve this, we used a lentivector carrying the ACE2 gene, successfully establishing the SH-ACE2 cell line. SH-SY5Y and SH-ACE2 cells were treated with CUR, EXT or curcuminoids (Me08 and Me23) for 2 h, after the treatment removal, the cells were infected with SARS-CoV-2 for 2 h. The virus was removed, and the RT-qPCR was performed after 24 h (Fig. 5A). Our findings revealed an upregulation of ACE2 expression in SH-ACE2, with levels exceeding 10,000 times that of SH-SY5Y cells (Fig. 5B, Unpaired t-test, t_4_ = 4.758, p < 0.05). This substantial ACE2 overexpression in SH-ACE2 corresponded with a significantly higher viral load, reaching 1.1X10^8^ PFU/mL at 24 h post-infection, representing a 4-log increase when compared to SH-SY5Y (Fig. 5C, One-way ANOVA, F_9,39_ = 123.4, p < 0.0001).

Furthermore, it's noteworthy that neither CUR, EXT, nor the curcuminoids Me08 and Me23 exhibited any significant effects on SARS-CoV-2 replication in SH-SY5Y cells. In contrast, both curcuminoids, Me08 and Me23, resulted in decreased viral loads, with a 1-log decrease and a substantial 3-log decrease, respectively, when compared to the CTL (Fig. 5C, One-way ANOVA, F_9,39_ = 123.4, p < 0.0001).

### CUR, EXT and curcuminoids ME08 and ME23 reduces the levels of INF-γ, TNF-α, IL-6 and IL-17 in SH-ACE2 cells infected with SARS-CoV-2

In light of the recognized role of inflammation in neuronal cell death and the potential neurotoxicity of various pro-inflammatory factors, including INF-γ, TNF-α, IL-6 and IL-17^44,45^, we sought to investigate the impact of CUR, EXT, and curcuminoids ME08 and ME23 on these factors in SH-ACE2 cells following SARS-CoV-2 infection.

**Figure 6:**
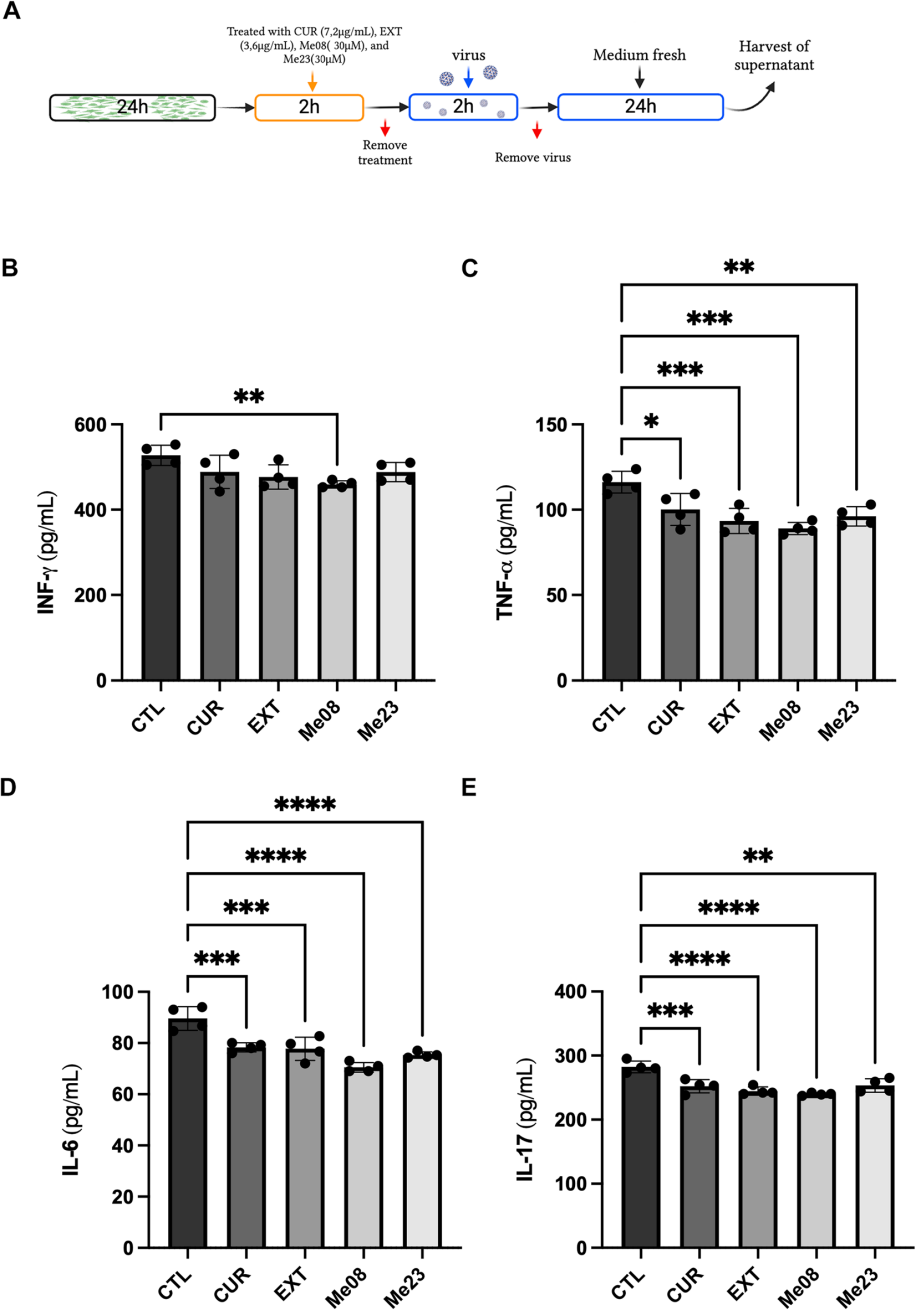
Cytokine’s expression after treatment with CUR, EXT, Me08 and Me23. (**A**) Cells were treated with CUR, EXT, Me08 or Me23 for 2 h, they were removed, and the cells were infected with SARS-CoV-2 (MOI 0.2) for 2 h, after viral removal, the supernatant was collected after 24 h and the cytokines’ expression was quantified by ELISA. (**B**) INF-γ, (**C**) TNF-α, (**D**) IL-6, (**E**) IL-17. CTL: Cells infected with SARS-CoV-2 and without treatment, CUR: curcumin, EXT: extract. *p < 0.05. (n = 4). The schematic diagram illustrating the infection process was based on a previous publication^67^.

To assess their effect on pro-inflammatory protein expression, we subjected SH-ACE2 cells to pretreatment with these compounds for 2 h. Subsequently, the cells were exposed to SARS-CoV-2 for 2 h, followed by the collection of supernatant after 24 h. We then quantified pro-inflammatory factors via ELISA (Fig. 6A).

Regarding INF-γ, Me08 exhibited a significant reduction of approximately 10% (459.4 ± 8.509 ng/mL) when compared to the CTL group (527.5 ± 23.54 ng/mL) (Fig. 6B, One-way ANOVA, F_4,15_ = 3.622, p = 0.0295).

Notably, all compounds demonstrated the capacity to decrease the levels of IL-6, TNF-α, and IL-17 (Fig. 6C,D,E). However, Me08 displayed a more pronounced anti-inflammatory effect among the compounds, resulting in reductions of approximately 25% for TNF-α, 20% for IL-6, and 15% for IL-17.

## Discussion

Studies investigating the utility and efficacy of CUR curcuminoids in various medical conditions, particularly in the context of COVID-19, have been gaining momentum during the ongoing pandemic. Recent clinical trials have yielded promising results, demonstrating the beneficial impact of CUR and curcuminoids on COVID-19. These effects encompass a reduction in time of hospitalization^46^, modulation of elevated inflammatory cytokine levels^47^, and expedited symptomatic recovery^48^. Understanding the devastating consequences of COVID-19, mainly on the central nervous system, we aimed to investigate the effects of CUR, EXT, and curcuminoids on neuronal cell line.

In our study we used CUR, obtained from a nutritional supplement capsule, EXT (*Curcuma longa*) and the curcuminoids, Me08 and Me23. We observed that concentrations of CUR exceeding 7.2 µg/mL exhibited cytotoxic effects in SH-SY5Y, reducing cell viability to less than 50%. In contrast, EXT and Me08 demonstrated no cytotoxicity within the concentrations studied. However, Me23 displayed cytotoxicity at concentrations above 60 µM. As a result, we opted to utilize CUR at 7.2 µg/mL, EXT at 3.6 µg/mL, and Me08 and Me23 at 60 µM.

Bormann et al. (2021) and Marín-Palma et al. (2021) used higher concentrations of CUR and EXT in their studies^49,50^. Nevertheless, it's important to note that they used VERO-E6 cells, a monkey cell line commonly used in SARS-CoV-2 replication studies, and also observed cytotoxic effects in the cells.

Once defined the non-cytotoxic concentrations of the compounds we have decided to investigate the possible effects of CUR, EXT and curcuminoids (Me08 and Me23) on the expression of key host receptors for the virus. A previous in silico study suggested that CUR may interact with the spike glycoprotein of SARS-CoV-2, impeding the virus-receptor interaction by binding to ACE2 residues^51^.

Our results did not reveal significant modulation in the gene or protein expression of ACE2 or furin. However, curcuminoids Me23 and Me08 demonstrated reductions in protease levels. Specifically, Me08 decreased the protein levels of TMPRSS11D, while Me23 reduced the gene expression of TMPRSS11D and the protein level of TMPRSS2. Recent studies have associated TMPRSS11D with enhanced SARS-CoV-2 entry^15,17^. Notably, TMPRSS11D is expressed in brain tissues (https://www.proteinatlas.org/ENSG00000153802-TMPRSS11D/brain, accessed on 15 October 2023), and overexpression of this protease in VERO-E6 cells has been shown to enhance SARS-CoV-2 entry^17^. Previous research has indicated that CUR decreased TMPRSS2 expression in prostate cancer cells, although this effect was not observed in SH-SY5Y cells^52^. It's also worth noting that Me23 is a newly explored curcuminoid that has not been tested in the context of COVID-19 until now.

Given the previous studies highlighting the antioxidative properties of CUR, EXT, and curcuminoids, we decided to investigate the levels of ROS following treatment with these compounds and SARS-CoV-2 infection. Our findings revealed that SARS-CoV-2 infection led to an increase in ROS levels in SH-SY5Y cells. This observation aligns with existing literature, as severe cases of COVID-19, where the virus penetrates the central nervous system (CNS), can trigger a cytokine storm. This, in turn, results in heightened ROS production and oxidative stress (OS). In response to OS, inflammatory cells release more pro-inflammatory cytokines, thereby exacerbating inflammation and intensifying ROS and OS. This creates a detrimental cycle of events^53–55^.These pro-inflammatory cytokines and OS can contribute to demyelination and axonal damage. Only Me23 reduced the levels of ROS to the MOCK levels. Curcumin's ability to cross the blood–brain barrier (BBB) is of particular interest since it can potentially reduce elevated ROS levels, protect the brain from lipid peroxidation, and decrease neuronal death due to oxidative damage^35^. Additional studies have observed a reduction in ROS levels following the administration of curcuminoids^56,57^, highlighting the antioxidative potential of these compounds. Contradictory results in our study might be attributed to differences in purity levels, concentration measurements, and the use of CUR dietary supplements.

We also decided to investigate the NRF2 expression, responsible for the body's antioxidant response^58^. A study using primary mouse osteoclast cultures revealed that treatment with pure CUR increased the expression of the NRF-2 gene^58^. NRF2 plays a crucial role in maintaining cellular homeostasis by regulating the expression of antioxidant enzymes and elements in the antioxidant response during oxidative stress (OS) processes^59^, such as heme oxygenase (HO-1) and NQO1, which mitigate OS progression and maintain redox balance, particularly in injured tissues and organic failure^60^. During infection, the SARS-CoV-2 can inhibit the NRF2 pathway, because the virus causes an exacerbated inflammatory response that contributes to the decrease of the NRF2 activity^60^, therefore, the virus causes an increase in OS and inflammatory disorder^61^. Ryan et al., 2022, observed that NRF2 can inhibit not only Interferon b but also IL-6 and TNF in inflammatory macrophages^62^. A clinical trial analyzed children with COVID-19 and observed a decrease in NRF2 levels in the children with COVID-19 when compared to the control group^63^. Some flavonoids e.g., curcumin, resveratrol, and naringenin, can decrease the OS through the NRF2 pathway^60^. Supporting this, Ding et al., 2022, analyzed a high-fat diet-fed mouse model treated with bisdemethoxycurcumin, observing increased NRF2 protein expression in the treated group compared with the control group. Additionally, in a study treating mice with traumatic brain injury with curcumin, an increase in both NRF2 and NQO-1 protein expression was observed compared to the control group^64^.

Our study aligns with these findings; we observed that the Me23 compound increased NRF2 gene expression in SH-SY5Y cells infected with SARS-CoV-2 compared with the control group. Additionally, we measured NQO1 activity and found that SARS-CoV-2 infection reduced NQO1 activity. However, in the presence of Me23, the activity was restored to levels similar to those observed in the MOCK. These results support the antioxidant role of curcuminoids such as Me23.

We further investigated the effects of CUR, EXT, and curcuminoids on SARS-CoV-2 replication. Previous studies involving SARS-CoV-2 infection in SH-SY5Y cells had shown limited viral replication^14^. To address this, we have decided to overexpress the ACE2 receptor in SH-SY5Y cells, thereby enhancing their capacity for extensive SARS-CoV-2 replication. Indeed, this genetic modification resulted in a viral load in SH-ACE2 cells that was 10,000 times higher than that in SH-SY5Y cells. Notably, the overexpression of the ACE2 receptor in SH-SY5Y cells revealed an antiviral effect of Me08 and Me23. It's worth mentioning that the antiviral effect of Me23 appeared to be more pronounced than that of Me08.

It has been demonstrated a reduction in viral replication in Calu-3 cells (human lung cancer cells) following treatment with EXT, CUR (in the form of a nutritional supplement capsule), and pure CUR^49^. They also reported a decrease in viral load in SARS-CoV-2-infected Vero-E6 cells when exposed to various concentrations of pure CUR. However, it's important to note that the authors used a concentration that was 10 times higher than the one we employed, and this higher concentration exhibited toxicity in our cells. To the best of our knowledge our study is the first to evaluate the antiviral effects of CUR, EXT and curcuminoids in neuronal cells.

Given the well-established anti-inflammatory properties of curcuminoids^40,65^, we further explored their role in modulating cytokines, including IL-6, IL-17, IFN-γ, and TNF-α, which are often elevated in COVID-19. Findings from other studies have shown increased cytokine levels in patients with neurological syndromes and COVID-19^22,24^. Interestingly, treatment with curcumin led to decreased gene expression of these inflammatory cytokines in peripheral blood mononuclear cells^50^. In alignment with these findings, our study indicated that all compounds (CUR, EXT, Me08, and Me23) reduced concentrations of IL-6, IL-17, and TNF-α, while Me08 specifically reduced IFN-γ levels in SH-ACE2 cells.

## Conclusion

**Figure 7:**
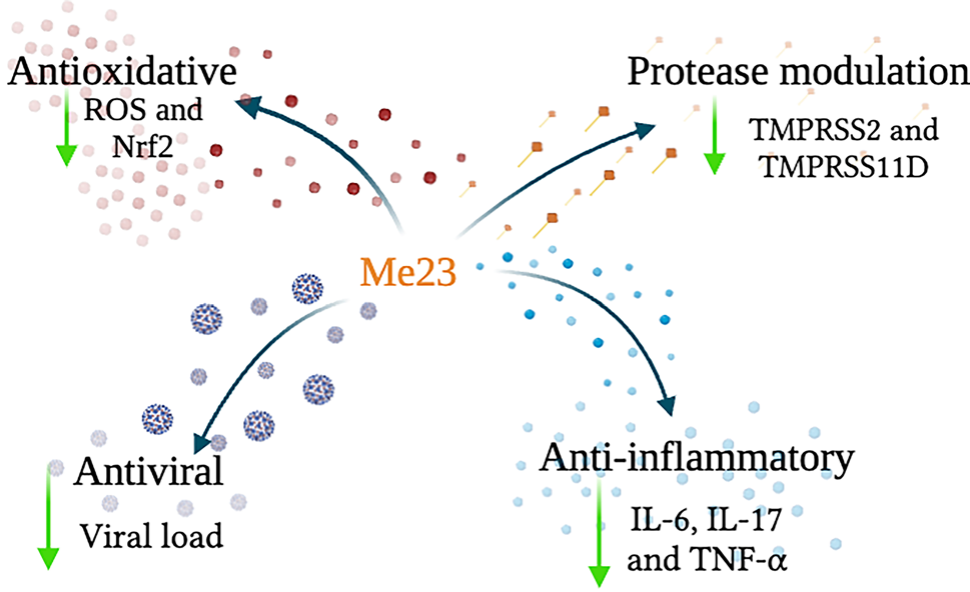
Schematic representation of the effects of Me23 in SH-SY5Y cells. Me23 (60 μM) reduced TMPRSS2, TMPRSS11D expression, ROS levels, viral load and decreased IL-6, IL-17 and TNF-α.

As summarized in Fig. 7, our study has revealed promising findings regarding the effects of curcuminoid Me23. We observed a reduction in TMPRSS2 and TMPRSS11D expression, a decrease in ROS levels induced by SARS-CoV-2, increase of the antioxidative pathway by modulation of NRF2 and NQO1, inhibition of SARS-CoV-2 replication, and noted anti-inflammatory effects in neuronal cells. These results not only contribute valuable insights into the potential of curcuminoids but also underscore the promising therapeutic prospects offered by Me23 in the context of COVID-19 treatment.

In conclusion, our study suggests that curcuminoid Me23 holds significant promise as an agent for mitigating the impact of COVID-19, particularly in the context of CNS involvement. Further research is warranted to delve deeper into its mechanisms of action and to assess its efficacy in clinical settings. These findings reinforce the importance of investigating natural compounds as potential allies in the battle against COVID-19 and related neurological complications.

## Methods

### Viral production and infection

Viral production was conducted in VERO-E6 cells (African green monkey kidney cells, ATCC CRL-1586), as previously described^66^. The cells were cultured in 10 cm plates with DMEM (Dulbecco's Modified Eagle's) without phenol red, supplemented with NaHCO_3_, 10% fetal bovine serum, and 0.02 mg/mL of gentamicin (Sigma Aldrich), hereafter referred to as cDMEM. The cells were maintained in a humid incubator at 37 °C in an atmosphere of 5% CO_2_. For plating, they were trypsinized and seeded in 24-well plates for the infection and treatment protocols. All in vitro experiments involving the propagation, titration, and infection of SARS-CoV-2 were carried out in the Biosafety Level III Laboratory (NB III) at the Biological Institute, in collaboration with Professor Dr. Líria Hiromi Okuda, following the regulatory recommendations of PAHO and WHO. The infection of SH-SY5Y cells followed the protocol previously described^16^. In summary, the cells were infected with SARS-CoV-2 isolated from a Brazilian patient (EPI_ISL_413016), using a multiplicity of infection (MOI) of 0.2 for 2 h at 37 °C. After infection, the cells were washed with PBS, and 500 µL of culture medium was added. After 24 h of infection, the viral supernatant was collected for RNA extraction, and the cells also had their total RNA extracted.

### Treatment with CUR, EXT, Me08 ad Me23

Concentration–response curves were generated to determine the optimal concentration and exposure time for CUR (BIOVEA CURCUMIN bcm-95®), EXT, Me08, and Me23 (Kindly donated by Dr Mirela Sairre, Federal University of ABC, Brazil). SH-SY5Y cells were cultured in 96-well plates (5 × 10^4^ cells/well) in 120 µl of cDMEM. Subsequently, the cells were treated with CUR at concentrations of 2.88 × 10^–2^ µg/mL, 1.44 × 10^–1^ µg/mL, 7.2 × 10^–1^ µg/mL, 3.6 µg/mL, 18 µg/mL, and 90 µg/mL for 24 h. EXT was used at concentrations of 1.44 × 10^–1^ µg/mL, 7.2 × 10^–1^ µg/mL, 3.6 µg/mL, 18 µg/mL, and 90 µg/mL, while Me08 and Me23 were tested at concentrations of 7.5 µM, 15 µM, 30 µM, 60 µM, and 90 µM for 24 h. After the treatment, cell viability was assessed using a colorimetric assay with 3-(4,5-dimethylthiazol-2-yl)-2,5-diphenyltetrazolium bromide (MTT) (Sigma-Aldrich, USA). Treated cells were incubated in 120 µl of DMEM supplemented with 10% FBS and 12 µl of 5 mg/ml MTT at 37 °C for 3 h. The formazan crystals formed were dissolved in 100 µl of dimethyl sulfoxide (DMSO). Optical density was measured at 590 nm in the spectrophotometer Spectramax M (Molecular Devices).

### Overexpression model of ACE2 in the SH-SY5Y cell line

The ACE2 overexpression model was developed in the SH-SY5Y cell line. SH-SY5Y cells were transduced with a lentiviral vector to overexpress ACE2 (pLENTI-hACE2-HygR, Addgene #161758). After 48 h of transfection, the cells were cultured in cDMEM for expansion and selection with hygromycin (80 μg/mL) for 14 days. Following this selection period, the cells were frozen for further experiments.

### RNA extraction and RT-qPCR

The gene expression of ACE2, TMPRSS2, TMPRSS11D and Furin, was assessed using RT-qPCR. Firstly, total RNA from the cells was extracted using Trizol reagent (Invitrogen) following the manufacturer's protocol. The purity and concentration of RNA were determined by absorbance using the NanoVue instrument (GE Healthcare). For the reverse transcription of RNA into cDNA, the High Capacity Kit (Applied Biosystems) was used. The reaction mixture consisted of 2 μg of RNA, 2 μL of 10X RT Buffer, 0.8 μL of 25X dNTP Mix (100 mM), 2 μL of 10X RT Random Primers, and 1 μL of the enzyme, and the volume was made up with water to achieve a final volume of 20 μL. The reaction conditions were as follows: 25 °C/10 min, 37 °C/120 min, and 85 °C/5 min.

Once the cDNA was obtained, the RT-qPCR reaction included 7.5 μL of SYBR-Green (Qiagen), 2 μL of cDNA (diluted 1/5 (v/v)), 1.2 μL forward primer, 1.2 μL reverse primer, 0.02 μL of ROX and 3.1 μL of water. The reaction was performed using the QuantiStudio 5 (Applied Biosystem). The following primers were used: ACE2 (ACE2_Fwd: 5′- TCC ATT GGT CTT CTG TCA CCCG -3′ ACE2_Rev: 5′- AGA CCA TCC ACC TCC ACT TCTC -3′), TMPRSS2 (TMPRSS2_Fwd: 5′-CCT CTA ACT GGT GTG A TG GCGT-3′ TMPRSS2_Rev: 5′-TGC CAG GAC TTC CTC TGA GATG-3′), TMPRSS11D (TMPRSS11D_Fwd: 5′-CAC AGT TCC AGA GCT AAG GCA-3′ TMPRSS11D_Rev: 5′-AAC CAA AGC CGC CGT GAG TCTT-3′), Furin (Furin_Fwd: 5′-ACT CCA CCT TGA GGT ACT CCA-3′ Furin_Rev: 5′-TAC GAG GGT GAA CTT GGT CAGC-3′), NRF2 (NRF2_Fwd:5′-CAC ATC CAG TCA GAA ACC AGT GG -3′NRF2_Rev: 5′-GGA ATG TCT GCG CCA AAA GCT G-3′) and RPL35 (RPL35_Fwd: 5′-CGA GTC GTC CGG AAA TCCAT-3′ RPL35_Rev: 5′-GGC TTG TAC TTC TTG CCC TTG-3′). The analysis was conducted using the relative comparison method (2^−ΔΔCT^), and the samples were compared to the internal control RPL35.

### ELISA

ELISA for INF-g, IL-6, IL-17, and TNF-a Cytokines were performed using cell culture supernatants collected 24 h after SARS-CoV-2 infection and compound treatment. Commercial ELISA kits (R&D Systems) were used following the manufacturer's protocol.

### CellRox

Cells were seeded in three 24-well plates (1 × 10^5^ cells/ well). After 24 h, they were treated for 2 h in DMEM (with 2% FBS without antibiotics) at 37 °C. Subsequently, the DMEM was removed, and the cells were washed with PBS. A new DMEM medium (with 2% FBS without antibiotics) containing the virus was added for 2 h at 37 °C. After this, the viral medium was removed, and fresh DMEM was added, followed by incubation in a 5% CO2 incubator at 37 °C for 24 h. In the last 30 min of incubation, 2.5 µM of CellROX™ Deep Red assay (#C10422, Thermo Fisher) was added to each well. Afterwards, the medium was discarded, and the cells were washed with PBS. The cells were trypsinized, and then DMEM (with 10% FBS without antibiotics) was added. All the liquid was transferred to 1.5 mL tubes. Subsequently, they were centrifuged at 1500 rpm for 5 min. After this, the supernatant was discarded, and the pellet was resuspended in PBS. Then, they were centrifuged again at 1500 rpm for 5 min. After that, the supernatant was discarded, and the cells were resuspended in 4% paraformaldehyde (PFA) and incubated for 20 min at 4 °C. After fixation, PBS was added, and the samples were taken for flow cytometry acquisition (FACS-AriaIII, BD Biosciences). Quantitative analysis of the acquisitions was performed using the FlowJo software.

### NQO1 activity

Cells were seeded in 24-well plates (1 × 10^5^ cells/ well). After 24 h, they were treated for 2 h with Me23 in DMEM (with 2% FBS without antibiotics) at 37 °C. Subsequently, the DMEM was removed, and the cells were washed with PBS. A new DMEM medium (with 2% FBS without antibiotics) containing the virus was added for 2 h at 37 °C. After this, the viral medium was removed, and fresh DMEM was added, followed by incubation in a 5% CO_2_ incubator at 37 °C for 24 h. After this, the cells were extracted using NQO1 ACTIVITY ASSAY KIT (ab184867, Abcam) following the manufacturer's protocol.

### Western blotting

For the analysis of ACE2, TMPRSS2, and TMPRSS11D protein expression, 5X 10^5^ cells were cultivated. After 24 h, they were treated for 24 h with Cur, Ext, Me08 and Me23 in DMEM-F12 supplemented with 10% FBS, under standard conditions (5% CO_2_, 37 °C). After incubation, supernatants were discarded, and adhered cells were washed with PBS1X. Cells were lysed with RIPA lysis buffer (150 mM NaCl; 1% NP-40; 0.5% deoxycholic acid; 0.1% SDS; 50 mM Tris pH 8.0; 0.2 mM MgCl_2_) and protease inhibitors. Total protein was extracted and quantified using a BCA kit (Thermo Fisher). Then, 20 μg of total protein from each strain were separated on 12% or 9% SDS-PAGE gels (according to the molecular weight of the protein of interest) and transferred to PVDF membranes.

The membranes were blocked with 5% bovine serum albumin (BSA) and incubated overnight at 4 °C with the primary antibody to the protein of interest (ACE2- ABCAM ‘ab108252’; TMPRSS2—Santa Cruz ‘sc515727’; and TMPRSS11D—GeneTex 'GTX117370'). The blots were then incubated with secondary antibodies labeled with horseradish peroxidase (HRP, Thermo Fisher). The blots were developed with ECL (Perkin Elmer, Waltham, Massachusetts, USA), and luminescence was recorded with a UVITEC digital photo documentation system (UVITEC Cambridge, Cambridge, Cambridgeshire, UK). Some membranes were stripped with Restore Plus Blot Buffer (Thermo Fisher) and processed again with a different primary antibody. The images were analyzed by densitometry using the Image J software (NIH, Bethesda, MD, USA), and data are presented as percentages, considering the sum of all densitometry values of each protein as 100%.

### Statistical analysis

The results were expressed as the mean ± SD (standard deviation). Data were evaluated first for normality using a Shapiro Wilk test. We used unpaired student’s t-test to compare the means of two-groups, one-way analysis of variance (ANOVA) with Tukey’s post-test for comparisons between three or more groups or Dunnet’s post-test for comparisons between three or more groups with the control. The significance level was set at 5% (p < 0.05). Statistical analyses were performed using GraphPad Prism version 9.0 (GraphPad Software, Inc., La Jolla, CA, USA). Sigma plot v13. (Systat, Palo Alto, CA, USA) was used to calculate the power of the test, only power > 0.8 was considered in the analysis.

### Supplementary Information

Supplementary Information.

## Data Availability

The datasets generated during and/or analyzed during the current study are available from the corresponding author on reasonable request.
