# Supplementary material for: Antiviral, anti-inflammatory and antioxidant effects of curcumin and curcuminoids in SH-SY5Y cells infected by SARS-CoV-2
Source: Sci Rep. 2024 May 10;14:10696. doi: 10.1038/s41598-024-61662-7 (PMC11087556; doi:10.1038/s41598-024-61662-7)
Supplement: Supplementary file 1 — Supplementary Information. [file 41598_2024_61662_MOESM1_ESM.pptx]

## Slide 1
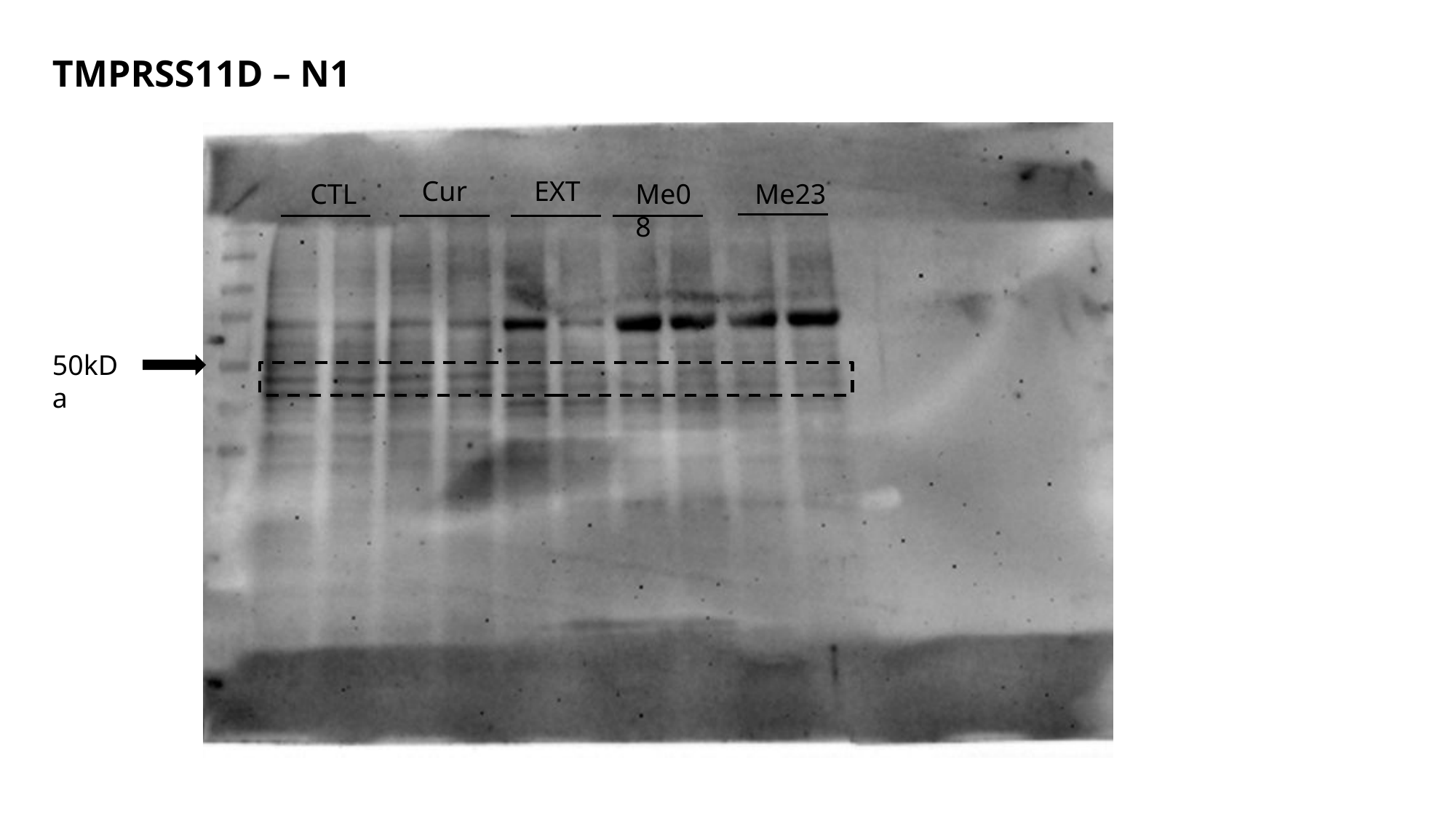

TMPRSS11D – N1
Cur
EXT
CTL
Me08
Me23
50kDa

## Slide 2
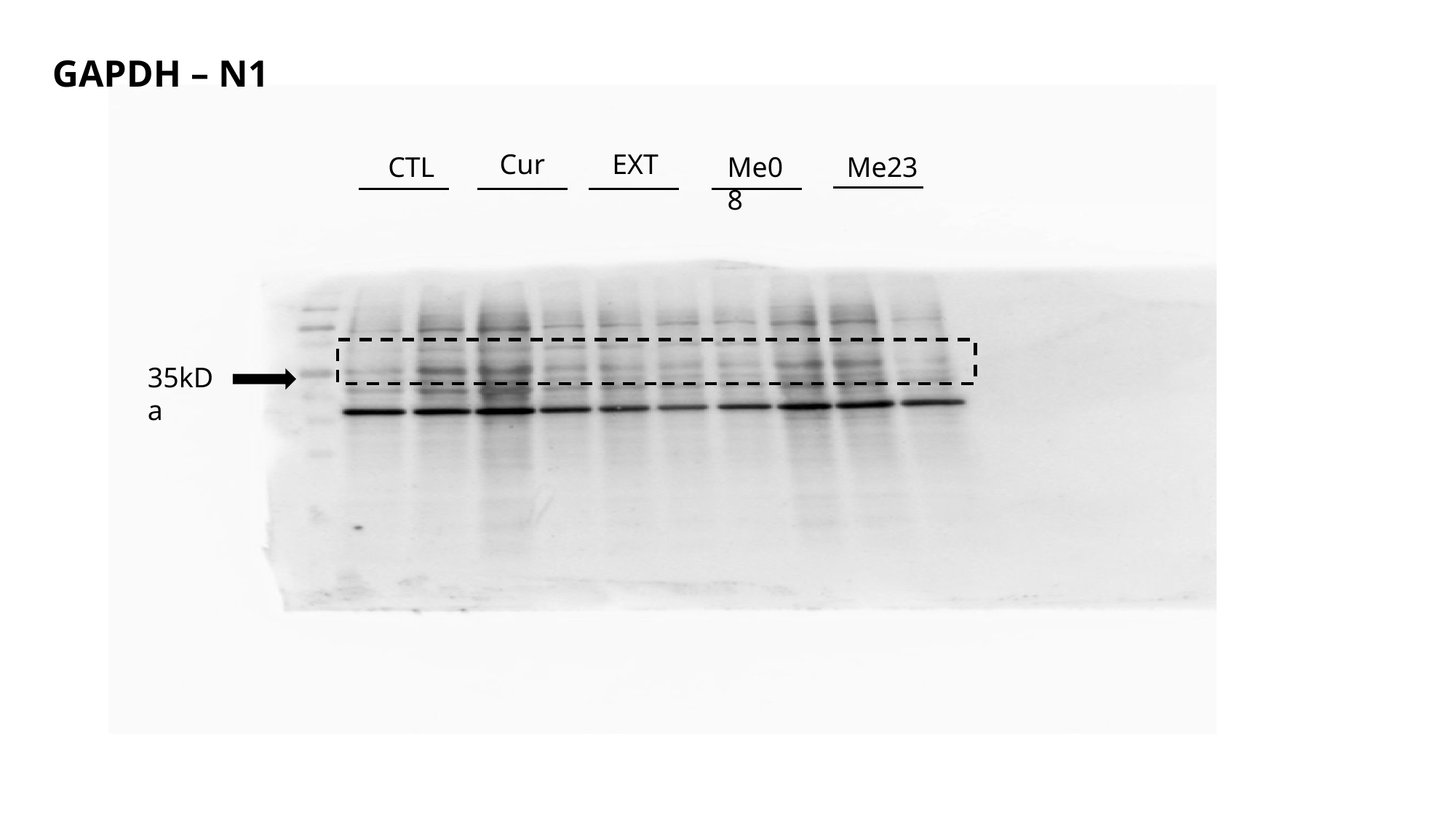

GAPDH – N1
Cur
EXT
CTL
Me08
Me23
35kDa

## Slide 3
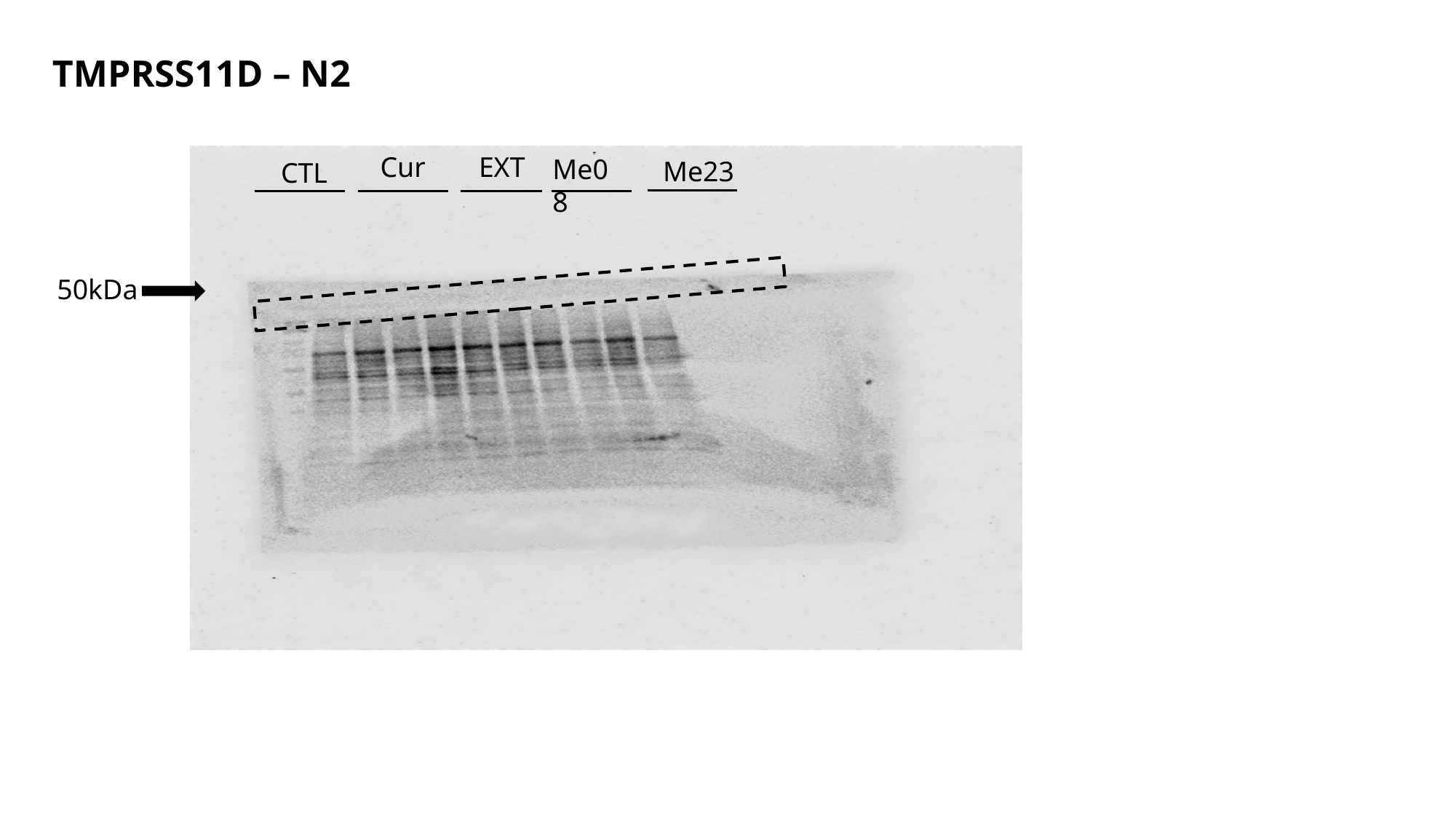

TMPRSS11D – N2
Cur
EXT
Me08
Me23
CTL
50kDa

## Slide 4
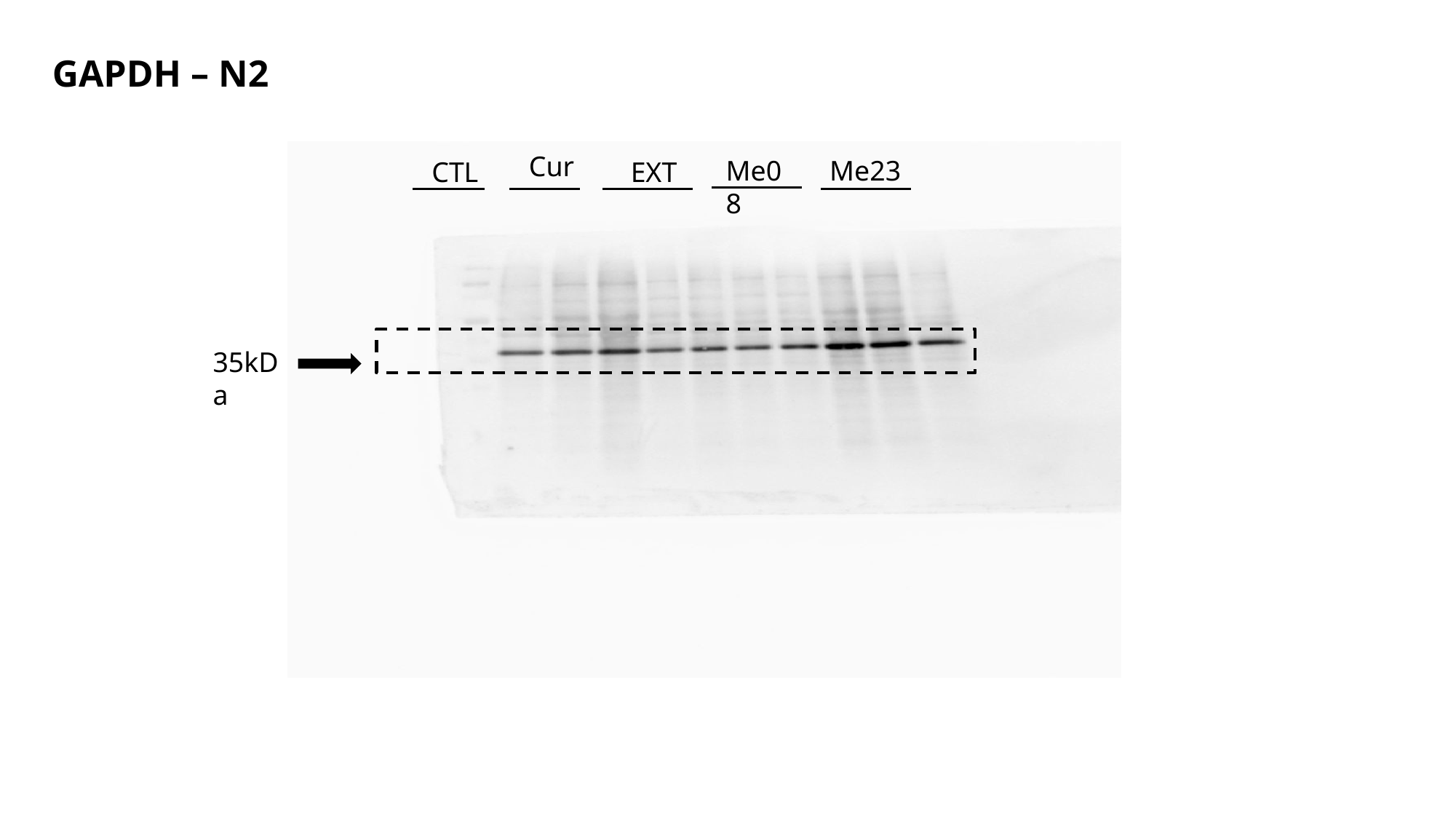

GAPDH – N2
Cur
Me23
Me08
CTL
EXT
35kDa

## Slide 5
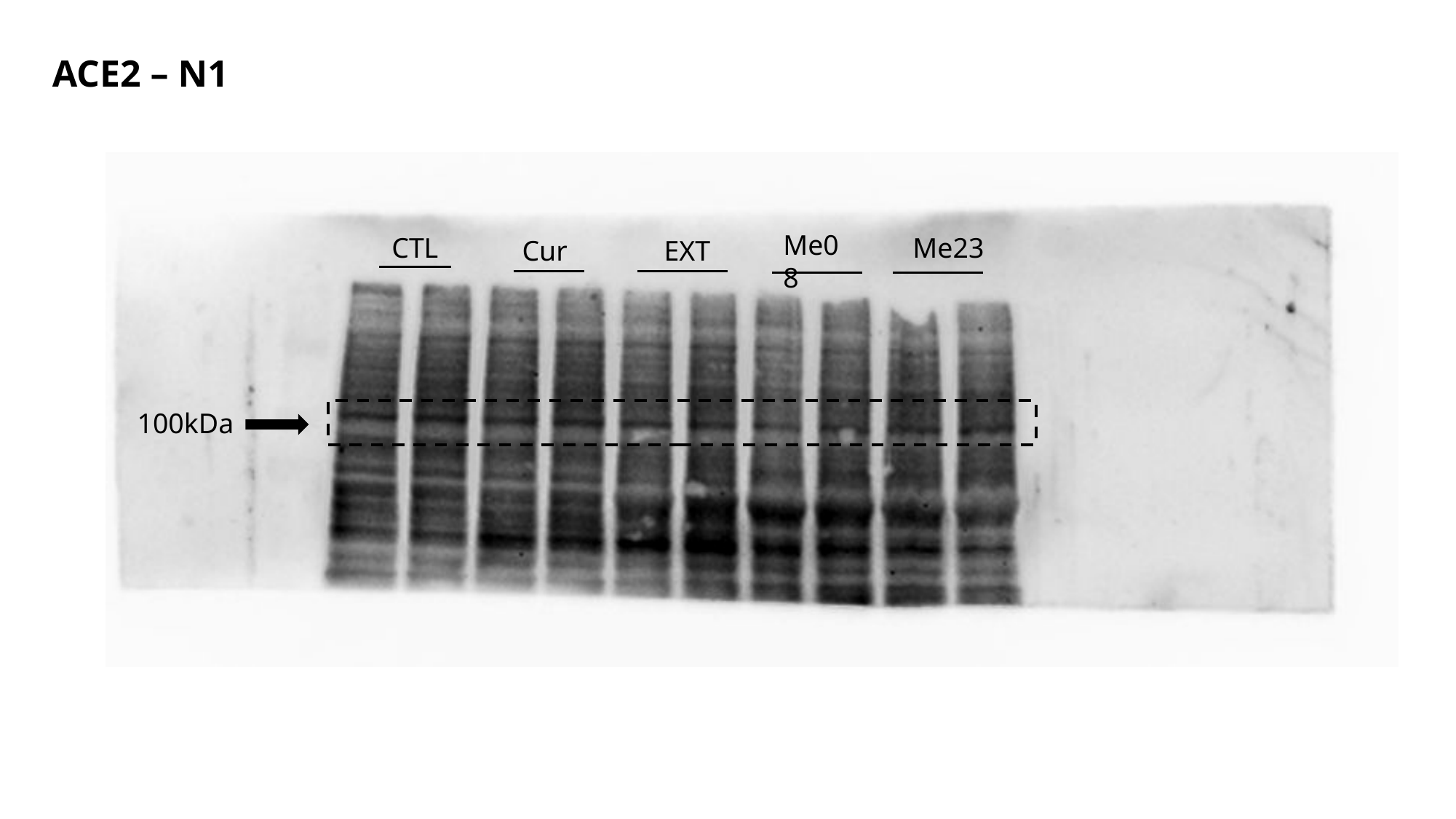

ACE2 – N1
Me08
CTL
Me23
Cur
EXT
100kDa

## Slide 6
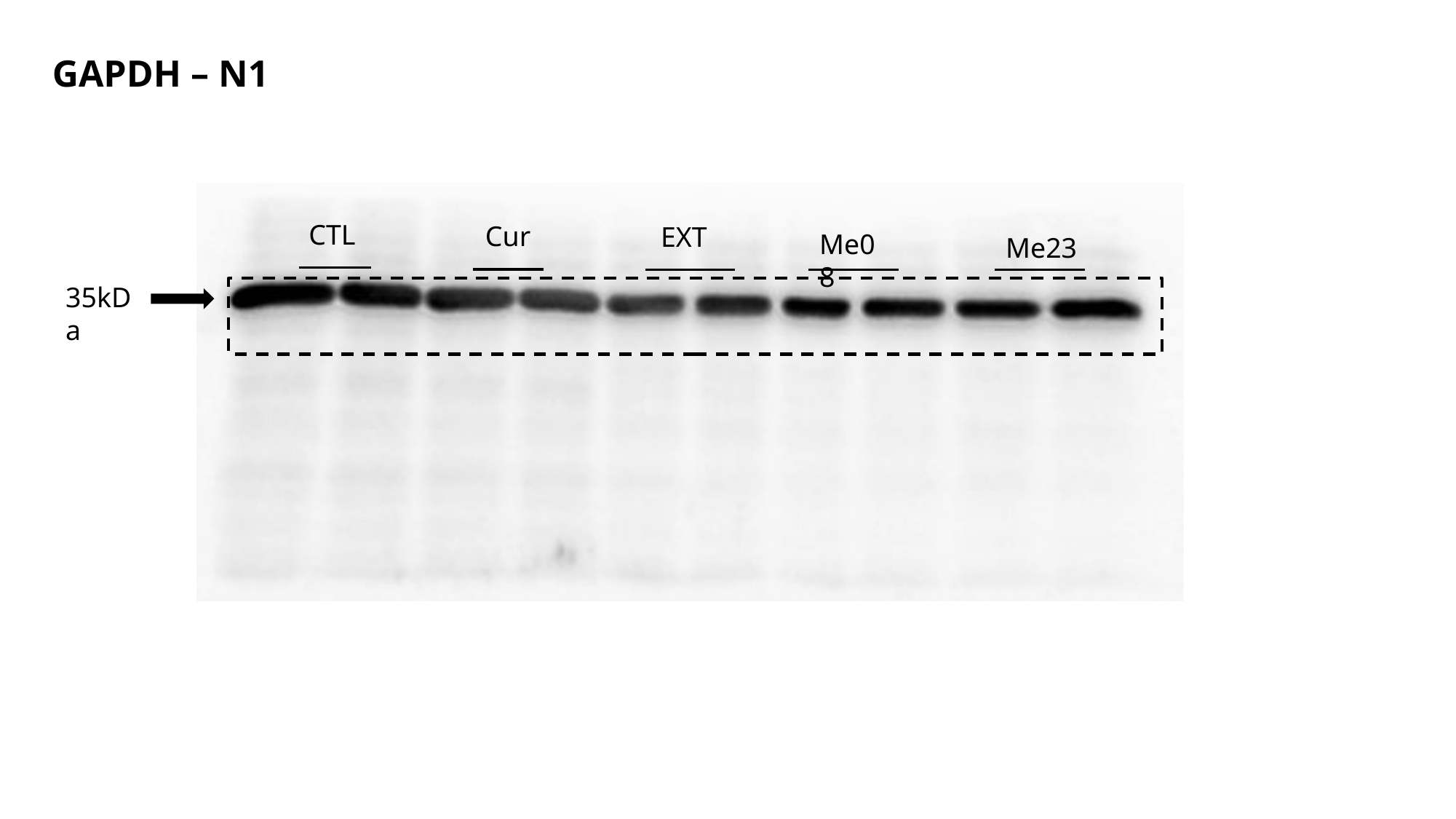

GAPDH – N1
CTL
Cur
EXT
Me08
Me23
35kDa

## Slide 7
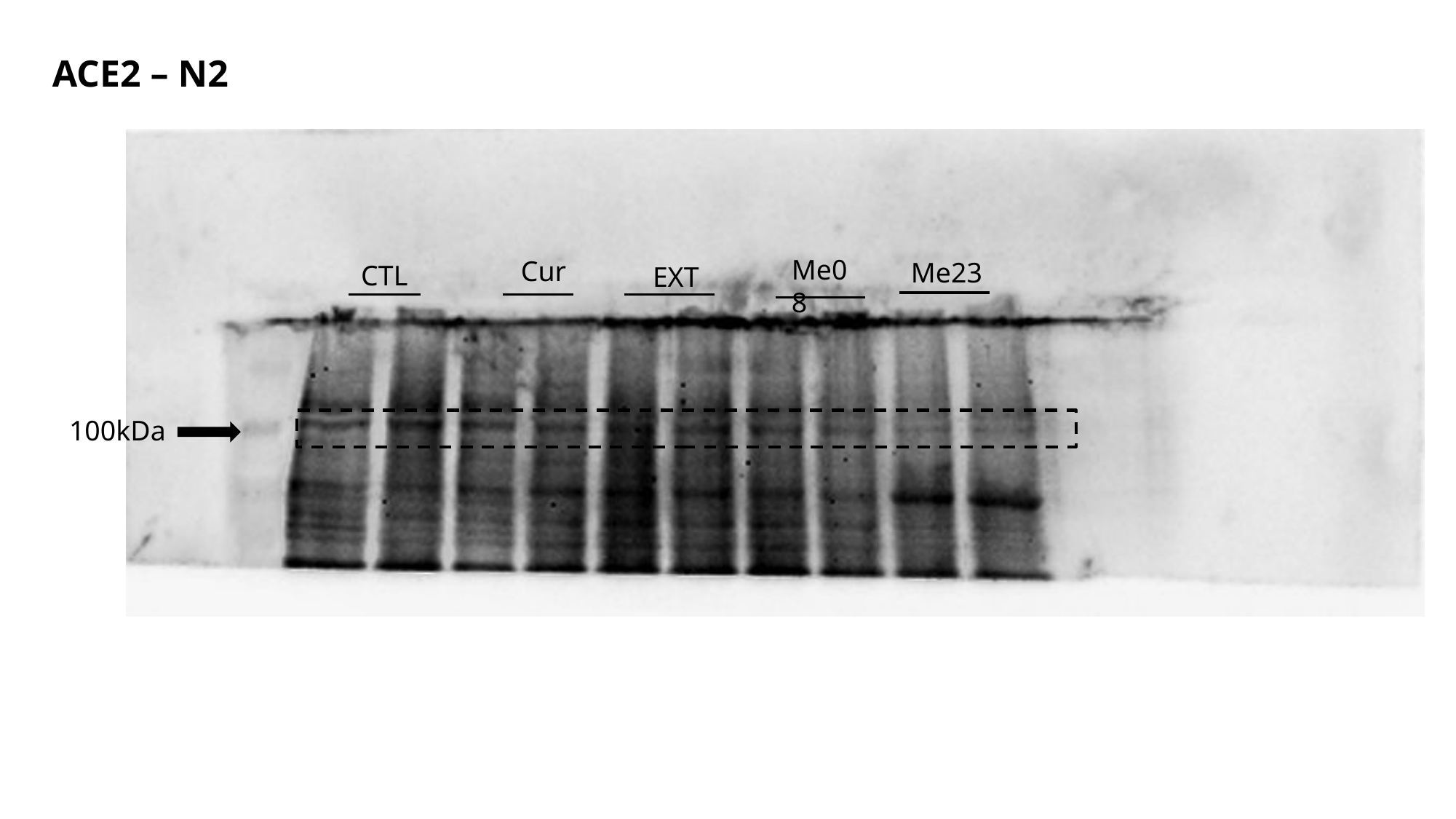

ACE2 – N2
Me08
Cur
Me23
CTL
EXT
100kDa

## Slide 8
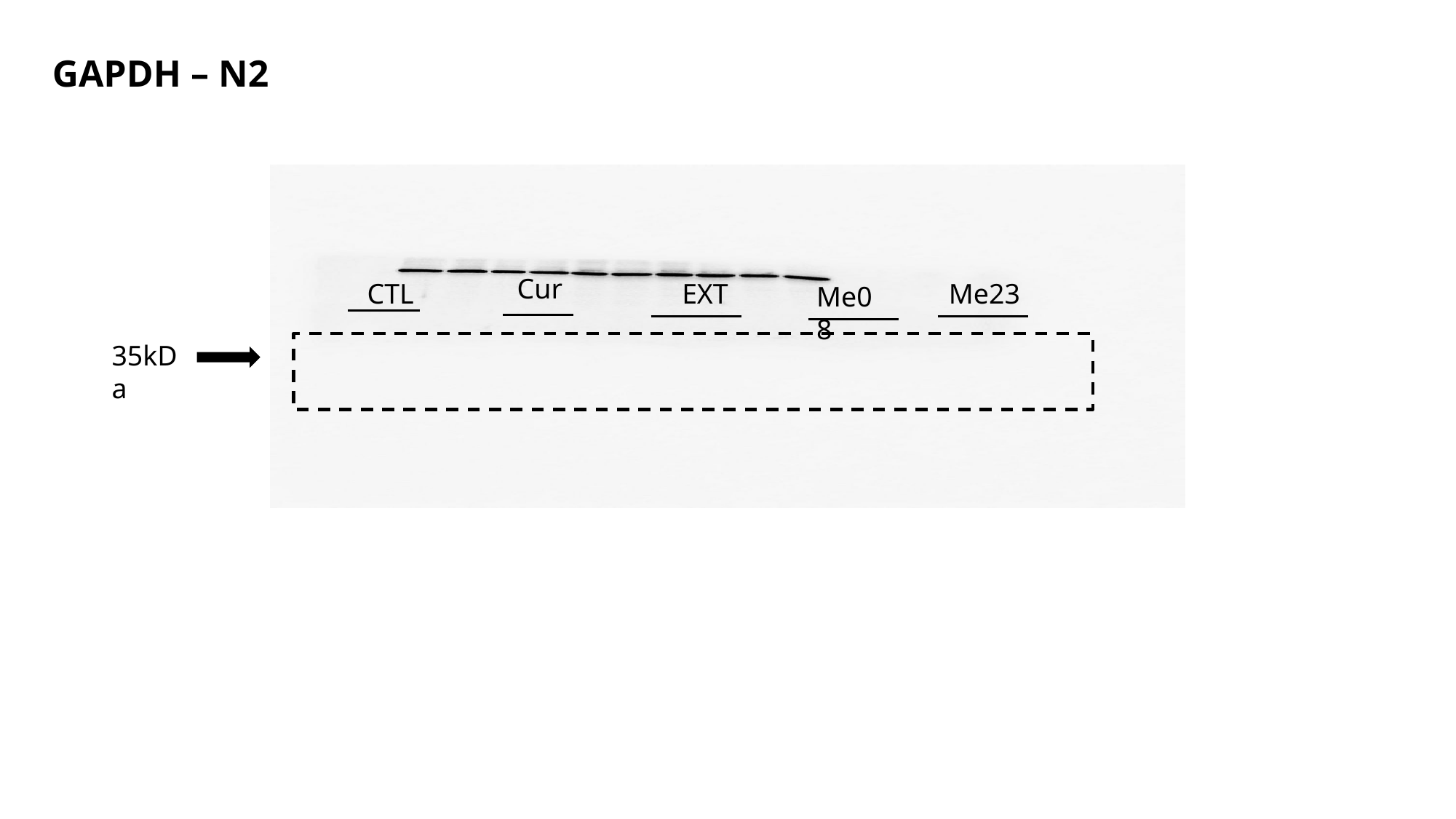

GAPDH – N2
Cur
CTL
EXT
Me23
Me08
35kDa

## Slide 9
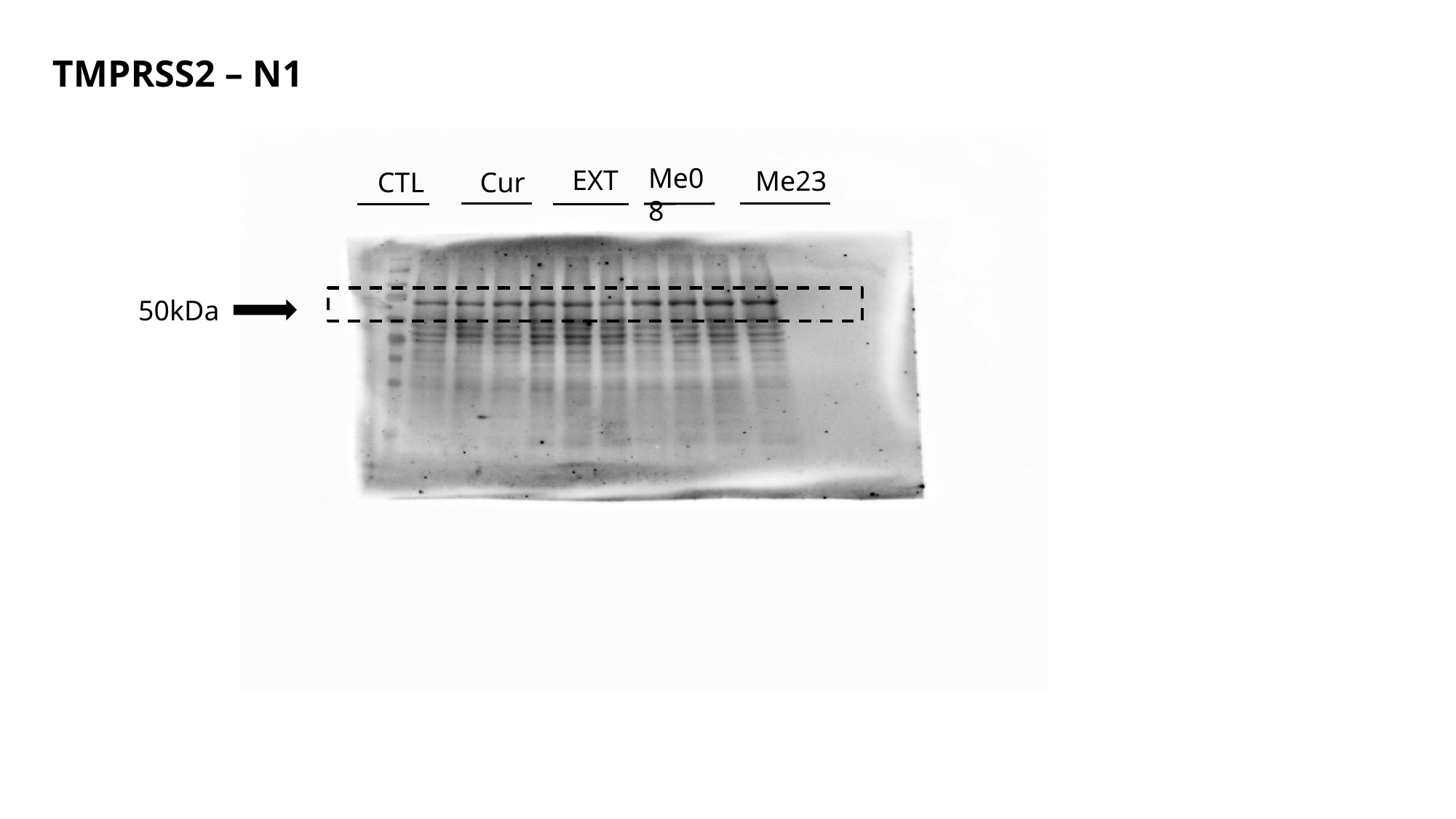

TMPRSS2 – N1
Me08
EXT
Me23
Cur
CTL
50kDa

## Slide 10
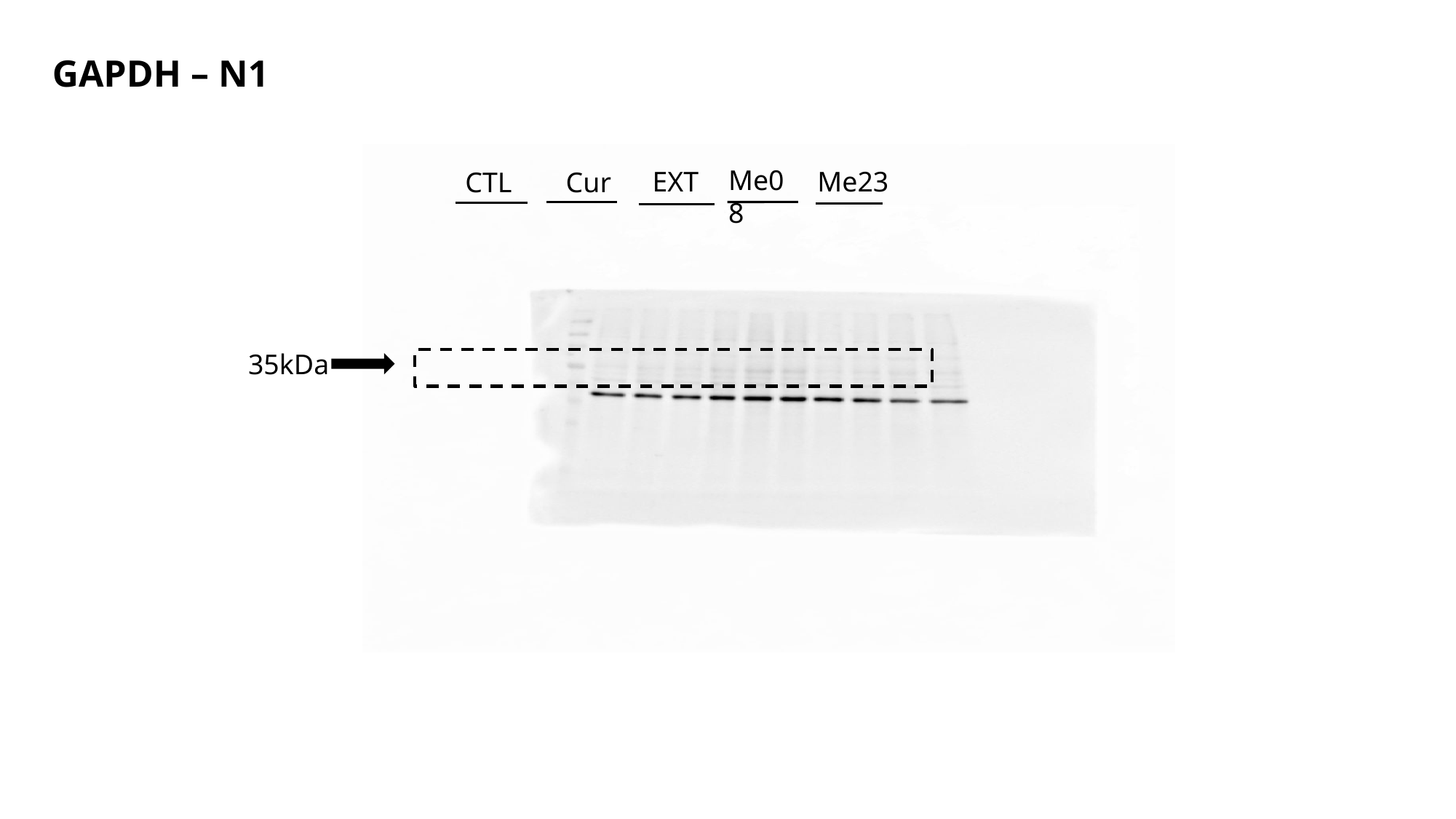

GAPDH – N1
Me08
Me23
EXT
CTL
Cur
35kDa

## Slide 11
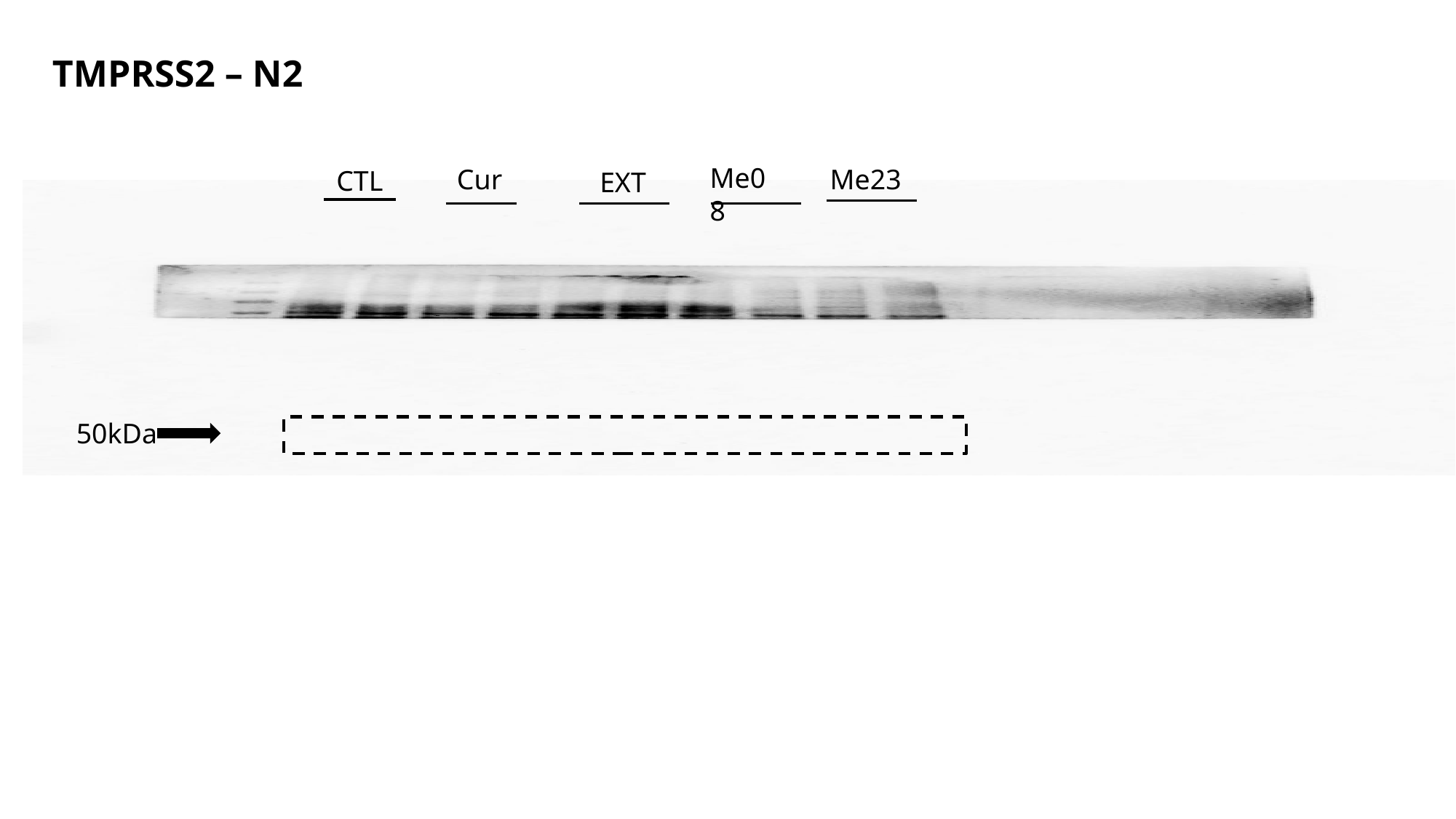

TMPRSS2 – N2
Me08
Me23
Cur
CTL
EXT
50kDa

## Slide 12
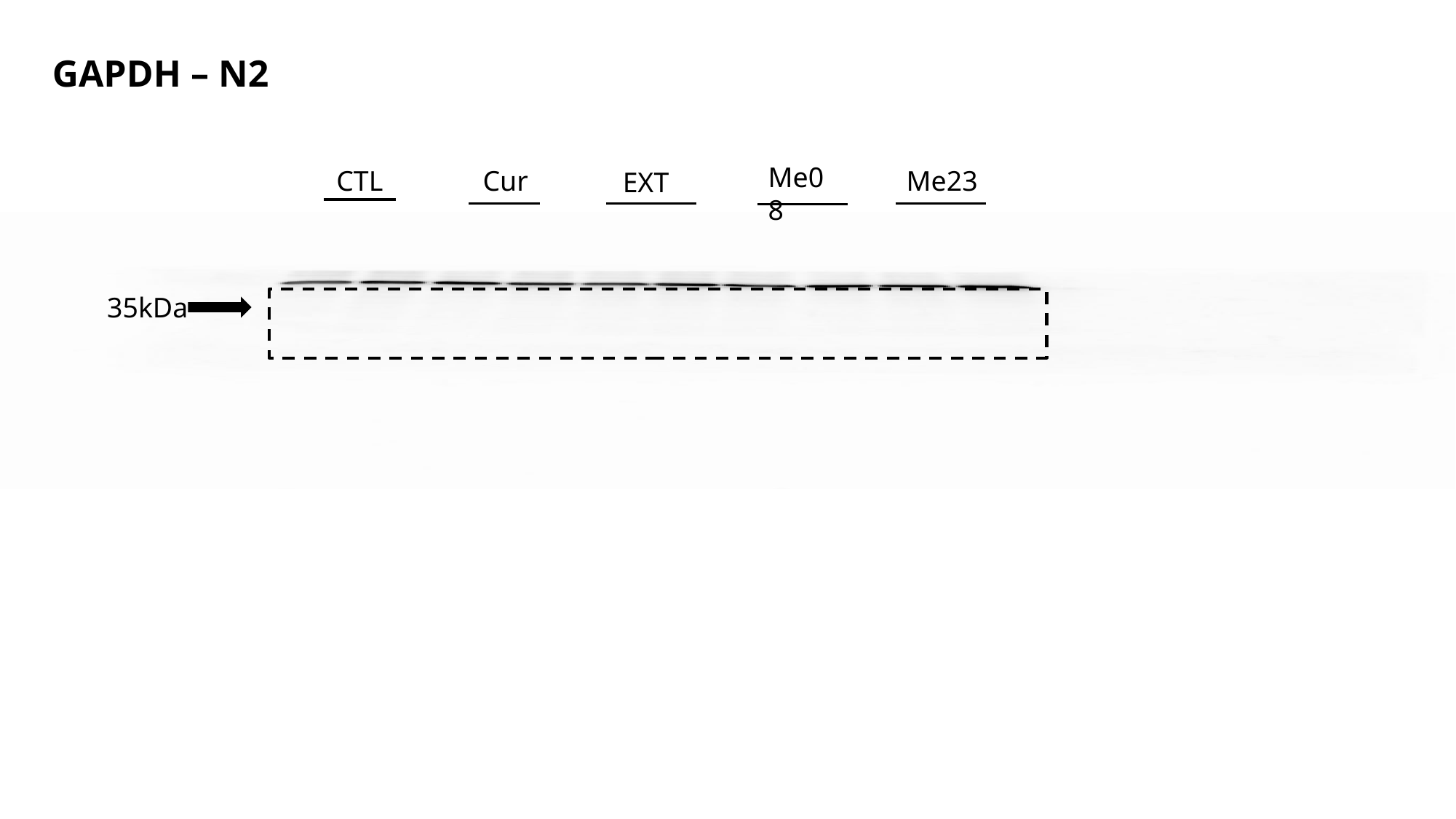

GAPDH – N2
Me08
CTL
Cur
Me23
EXT
35kDa
